# Determinants of diabetic nephropathy among diabetic patients in Ethiopia: Systematic review and meta-analysis

**DOI:** 10.1371/journal.pone.0297082

**Published:** 2024-02-02

**Authors:** Abere Woretaw Azagew, Zerko Wako Beko, Chilot Kassa Mekonnen

**Affiliations:** Department of Medical Nursing, School of Nursing, College of Medicine and Health Sciences, University of Gondar, Gondar, Ethiopia; Wollega University, ETHIOPIA

## Abstract

**Introduction:**

Diabetic nephropathy (DN) is a long-term kidney disease among diabetic patients. It is the leading cause of end-stage renal failure. In Ethiopia, DN affects the majority of diabetic populations, but there were inconsistent findings about the determinant factors across the studies.

**Methods:**

We have accessed studies using PubMed, Embase, EBSCO, Web of Science, OVID, and search engines including Google and Google Scholar published up to June 2023. The study populations were diabetic patients with nephropathy. The quality of each included article was assessed using the Newcastle-Ottawa quality assessment scale. The odds ratios of risk factors were pooled using a random-effect meta-analysis model. Heterogeneity was assessed using the Cochrane Q statistics and I-Square (I^2^). The publication bias was detected using the funnel plot and/or Egger’s test (p< 0.05). Trim and fill analysis was carried out to treat the publication bias. The protocol has been registered with the reference number CRD42023434547.

**Results:**

A total of sixteen articles were used for this reviewed study. Of which, eleven articles were used for advanced age, ten articles for duration of diabetic illness, ten articles for poor glycemic control, and eleven articles for having co-morbid hypertension. Diabetic patients with advanced age (AOR = 1.11, 95% CI: 1.03–120, I^2^ = 0.0%, p = 0.488), longer duration of diabetic illness (AOR = 1.23, 95% CI = 1.05–1.45, I^2^ = 0.0%, p = 0.567), poor glycemic control (AOR = 2.57, 95% CI: 1.07–6.14; I^2^ = 0.0%, p = 0.996), and having co-morbid hypertension (AOR = 4.03, 95% CI: 2.00–8.12, I^2^ = 0.0%, p = 0.964) were found to be factors associated with DN.

**Conclusions:**

The findings of the study revealed that diabetic patients with advanced age, longer duration of diabetic illness, poor glycemic control status, and co-morbid hypertension were the determinant factors of DN. Therefore, treatment of co-morbid hypertension and high blood glucose and regular screening of renal function should be implemented to detect, treat, and reduce the progression of DN. Furthermore, healthcare workers should give due attention to diabetes with advanced age and a longer duration of diabetes illness to prevent the occurrence of DN.

## Introduction

Diabetes mellitus is a major public health problem worldwide. The global prevalence was 10.5% in 2021 and has been projected to be 12.2% in 2045 [1]. In Ethiopia, it ranged from 2.0% to 6.5% [2]. The mortality and morbidity of diabetes mellitus are associated with chronic complications, such as vascular and non-vascular. Diabetic nephropathy (DN) is one of the major micovascular complications that leads to end-stage renal disease [3,4]. It is the leading public health challenge globally. Diabetic nephropathy is also known as diabetic kidney disease (DKD). Clinically, DN is characterized by an increase in urine albumin excretion (microalbuminuria) and/or a decreased glomerular filtration rate (GFR) [5–7]. It is the only complication for diabetic populations compared with non-diabetics [8]. The causes of DN are multifactoral, but they are associated with being overweight, diet, exercise, smoking, co-morbidity, type of diabetes, and non-adherence to diabetic medications [5,7,9–14].

Diabetic nephropathy is increasing significantly in low- and middle-income countries, while the remaining is under-recognized as a global illness burden [6,15]. The median time for diabetic patients to develop DN was 94.9 months [10]. Its prevalence was 21.8% in China [12] and 10.8% in Saudi Arabia [14]. Overall, DN occurs in 20–40% of all types of diabetics [16].

Diabetes and its complications affect individuals, families, healthcare systems, and national economies [17]. The World Health Organization-led initiative is targeted at reducing diabetic-associated kidney disease by providing comprehensive care [18]. Preventive strategies improve glycemic control by adhering to medication, food, and exercise [5]. Early detection and screening for microalbuminuria should be done regularly [6,7]. In advanced cases, patients require renal replacement therapy (RRT), such as hemodialysis, peritoneal dialysis, or kidney transplantation [19].

Though various studies have been undertaken on DN, the knowledge of the determinant factors of DN has been inconsistent across studies among diabetic patients. Therefore, this review study aimed to investigate the determinants of DN among diabetic patients in Ethiopia.

## Methods

### Study protocol registration and reporting

The review protocol has been registered with reference number CRD42023434547. The result of the review was presented based on the standard of preferred reporting items for systematic review and meta-analysis (PRISMA-2020) checklist [20] (**S1 File**).

### Searching strategies

The procedure for this study was designed following the PRISMA guidelines [20]. We search on electronic databases such as PubMed, Embase, EBSCO, Web of Science, OVID, and search engines (Google and Google Scholars) for grey literature. Endnote (version 7) reference management software was used to export, download, organize, review, de-duplicate, and cite the articles. The search strategies were carried out using controlled vocabulary (MeSH) terms. The synonym for diabetic nephropathy was identified. Then, the search string was established using the databases. Articles were searched by title (ti), abstract (ab), and/or full-text (ft). Boolean logic operators “AND” and “OR” were used to combine the search terms. The search string was expressed as “diabetic nephropath*” OR “diabetic kidney disease” OR DKD OR “chronic kidney disease” OR CKD OR “diabetic glomerulosclerosis” OR “Kimmelstiel-Wilson disease” OR “Kimmelstiel-Wilson Syndrome” OR “nodular glomerulosclerosis” AND “diabetic mellitus” OR “Type 1 diabetic mellitus” OR “T1DM” OR “Type 2 diabetic mellitus” OR T2DM, OR Hyperglycemia, OR “elevated blood glucose” AND Ethiopia. This search included articles published up to June 2023.

The search limiters, such as study design, country of the study, and language of publication, were used. Furthermore, to include additional relevant articles, citation tracking and personal contact with experts were carried out. Two reviewers (ZWB and CKM) screened articles by title (ti), abstract (ab), and full-text (ft). The disagreements between the reviewers were resolved by discussion.

### Study population

The populations for this systematic review and meta-analysis were diabetic patients with nephropathy.

### Inclusion and exclusion criteria

In this systematic review and meta-analysis, articles that fulfill the eligibility criteria: (1) articles with observational studies such as cross-sectional, cohort, and case-control; (2) articles that report the determinants, risk factors, or associated factors of diabetic nephropathy; (3) studies conducted in Ethiopia; (4) both published and/or unpublished articles; (5) articles published or conducted up-to June 2023; (6) articles written in English; and (7) articles conducted in community or health facility settings were included in the study. Articles with trials, without full text, conference papers, and systematic reviews and meta-analyses were excluded from the study.

### Quality appraisal

The quality of the study was evaluated using the Newcastle Ottawa Quality Assessment Scale (NOS), adapted from the cross-sectional [21], cohort [22], and case-control [23] studies. Two reviewers (ZWB and CKM) independently reviewed the quality of the included studies. The inconveniences between the reviewers were resolved by discussion. A score of ≥7 was considered a high-quality score [24].

### Outcome measurements

Diabetic nephropathy is defined as a derangement in renal function with an estimated GFR <60 ml/min/1.73 m^3^, and kidney damage, usually by estimation of albumin uria >30 mg/dl [25].

### Data extraction

After the quality assessment of the studies, the data were extracted using the Microsoft Excel speed sheet. The extracted data item includes authors, publication year, study design, region of the study, data collection method, funding source, and other effect measures (**S2 File**). Two reviewers (ZWB and CKM) independently extracted the data. The disagreements between the reviewers were resolved through discussion and third-reviewer involvement.

### Data analysis

The extracted data were entered into a Microsoft spreadsheet and exported to Stata version 14 for analysis. The heterogeneity of the studies was assessed using the I-squared (I^2^) and Cochrane Q statistics with a p-value > 0.05. The I^2^ test statistics result of 0.0%, 25%, 50%, and >75% were considered as no, low, moderate, and considerable heterogeneity [26], respectively. A random-effect meta-analysis model [27] was used to estimate the effect of determinant factors on diabetic nephropathy. The forest plot was used to visualize the pooled effect size with the respective odds ratio and 95% confidence interval. The publication bias was detected by the visual inspection of the graphic asymmetry test of the funnel plot [28] and/or Egger’s test (P<0.05) [29]. Trim and fill analysis was computed to treat the publication bias [30].

## Results

### Study selection and characteristics

**Fig 1:**
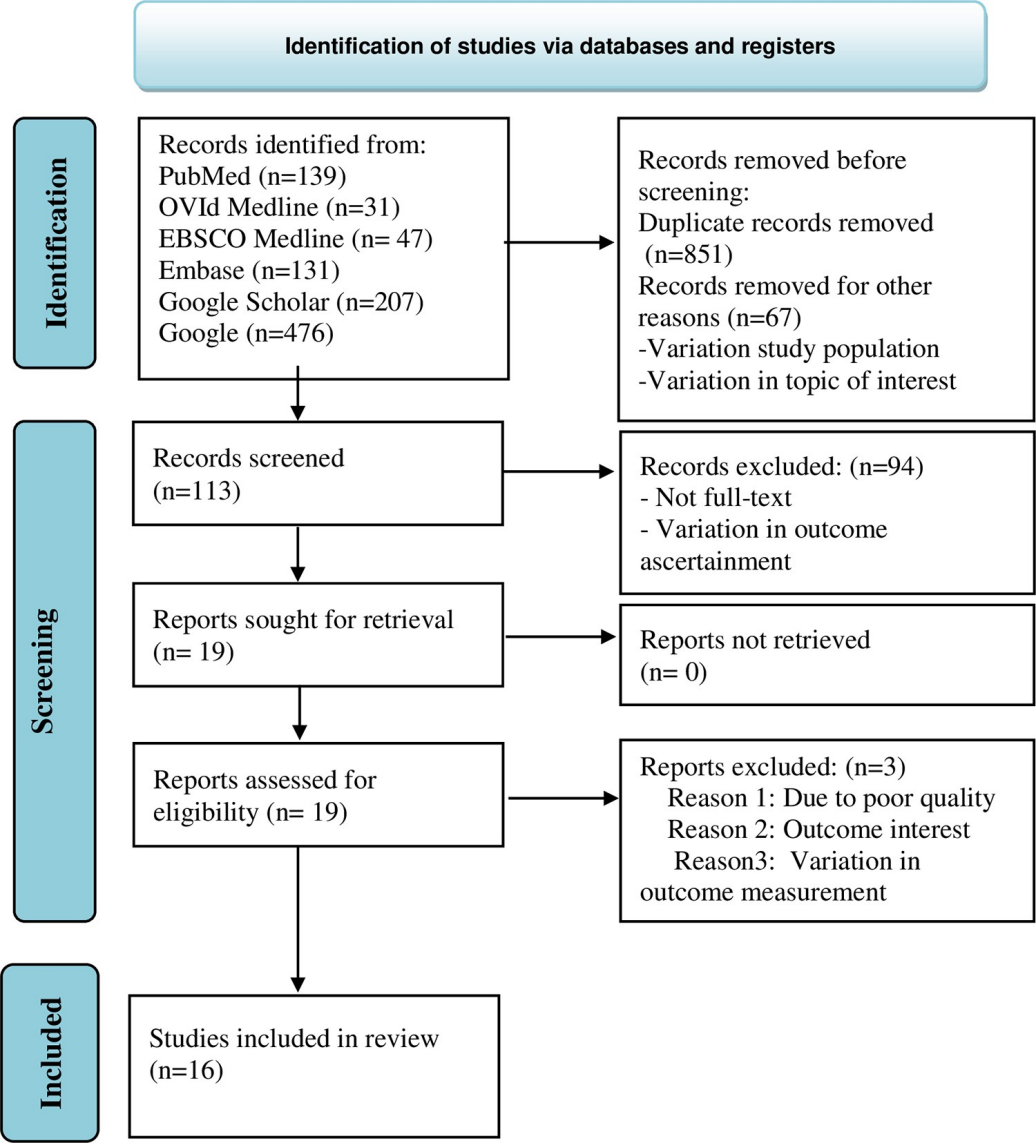
PRISMA flow diagram for the flow of information through the phases of the review.

The search strategy retrieved 1031 both published and/or unpublished original articles. After the removal of duplicate articles, 113 articles remained. Following further screening, about 19 articles were assessed for eligibility, of which three were excluded because of reporting without the outcome of interest (the DN is not clearly defined) and poor quality (the NOS score <5) **(Fig 1)**. Finally, sixteen articles were used to extract the determinant factors of DN. Of these, eleven articles were retrieved for age of diabetic patients [5,9,31–39] (**Table 1**), ten for duration of diabetic illness [5,9,15,31,32,36,38–41] **(Table 2)**, ten for poor glycemic control [5,9,32–34,36,39,41–43] (**Table 3)**, eight for elevated systolic blood pressure [9,15,33,34,36,38,41,44] **(Table 4)**, and twelve for co-morbid hypertension [5,15,31,33,36,38–44] (**Table 5)**. From the included studies, seven were in the Amhara region [15,31,34,38,39,41,44], four in the Oromia region [33,36,37], two in Addis Ababa [40,42], two in the Tgray region [5,9], and two in the SNNP region [32,43] of Ethiopia. Regarding the study design, as shown in each table, most of the studies were cross-sectional studies. The majority of the studies used both patient interviews and record reviews for data collection. Most of the included studies were not funded or their funding statuses were unreported. The average quality appraisal score of the included studies was ≥7.

**Table 1:** Study characteristics for the age of diabetic patients in Ethiopia.

Authors	year	study design	Region	Odds ratio	95% CI	quality score
Damtie S, et al. [31]	2018	Cross-sectional	Amhara	5.239	2.255–12.175	9
Fiseha T, et al. [32]	2014	Cross-sectional	SNNP	5.3	1.81–15.56	8
Dinku B, et al. [33]	2022	Cross-sectional	Oromia	2.17	1.09–4.31	8.5
Hintsa S, et al. [9]	2017	Case-control	Tigray	1.037	1.01–1.064	7
Mulu GB, et al. [34]	2023	Cross-sectional	Amhara	4.1	2.2–7.7	7.5
Zemichael TM, et al. [5]	2020	Case-control	Tigray	1.19	1.16–1.23	8
Abdulkadir M, et al. [35]	2022	Cross-sectional	Addis Ababa	5.8	1.5–21	8
Adem M, et al. [36]	2017	Cross-sectional	Oromia	3.02	1.55–5.9	7.5
Goro KK, et al. [37]	2019	Cross-sectional	Oromia	2.01	1.1–5	8.5
Tesfe D, et al. [38]	2022	Cross-sectional	Amhara	5.74	3.05–10	9
Fiseha T, etal. [39]	2020	Cross-sectional	Amhara	2.48	1.13–5.43	8

Notes:- **CI-Confidence interval; St PMMC-** Siant Paulos Millennium Medical College; **SNNP**-Southern Nations, Nationalities and Peoples.

**Table 2:** Study characteristics for the duration of diabetic illness among diabetic patients in Ethiopia.

Authors	year	study design	Region	Odds ratio	95% CI	quality score
Alemu H, et al. [15]	2020	Cross-sectional	Amhara	3.2	2.0–7.0	9
Bekele MM, et al. [40]	2016	Cross-sectional	Addis Ababa	1.45	0.73–2.88	8
Damtie S, et al. [31]	2018	Cross-sectional	Amhara	3.38	1.393–8.198	9
Fiseha T, et al. [32]	2014	Cross-sectional	SNNP	4.08	1.7–9.77	8
Hintsa S, et al. [9]	2017	Case-control	Tigray	1.13	1.077–1.18	7
Taderegew MM, et al. [41]	2020	Cross-sectional	Amhara	3.38	1.45–7.82	8
Zemichael TM, et al. [5]	2020	Case-control	Tigray	1.83	1.62–2.06	8
Adem M, et al. [36]	2017	Cross-sectional	Oromia	4.44	2.3–8.57	7.5
Tesfe D, et al. [38]	2022	Cross-sectional	Amhara	3.37	1.86–6.1	9
Fiseha T, et al. [39]	2020	Cross-sectional	Amhara	5.16	1.13–12.51	8

**Notes:- CI-** Confidence interval; **SNNP**-Southern Nations, Nationalities and Peoples.

**Table 3:** Study characteristics for patients with poor glycemic status among diabetic patients in Ethiopia.

Authors	year	study design	Region	Odds ratio	95% CI	quality score
Aberra T, et al. [42]	2022	Cross-sectional	Addis Ababa	1.23	0.667–2.276	9
Fiseha T, et al. [32]	2014	Cross-sectional	SNNP	4.65	1.69–12.76	8
Dinku H, et al. [33]	2022	Cross-sectional	Oromia	1.37	0.75–2.49	7.5
Hintsa S, et al. [9]	2017	Case-control	Tigray	2.71	1.49–4.95	7
Mulu GB, et al. [34]	2023	Cross-sectional	Amhara	2.5	1.5–4.1	7.5
Taderegew MM, et al. [41]	2020	Cross-sectional	Amhara	2.82	1.13–7.05	8
Zemichael TM, et al. [5]	2020	Case-control	Tigray	3.27	1.31–8.31	8
Adem M, et al. [36]	2017	Cross-sectional	Oromia	7.24	2.16–24.22	7.5
Nibret E, et al. [43]	2020	Cohort	SNNPR	9.09	4.0–20.0	8.5
Fiseha T, et al. [39]	2020	Cross-sectional	Amhara	2.58	1.19–5.61	8

**Notes: CI-** Confidence interval; **SNNP**-Southern Nations, Nationalities and Peoples.

**Table 4:** Study characteristics for patients with elevated systolic blood pressure among diabetic patients in Ethiopia.

Authors	year	study design	Region	Odds ratio	95% CI	quality score
Dinku B, et al. [33]	2022	Cross-sectional	Oromia	3.2	1.36–7.51	8.5
Hintsa S, et al. [9]	2017	Case-control	Tigray	2.78	1.69–4.58	7
Mulu GB, et al. [34]	2023	Cross-sectional	Amhara	1.9	1.0–3.4	7.5
Taderegew MM, et al. [41]	2020	Cross-sectional	Amhara	0.78	0.49–1.07	8
Alemu H, et al. [15]	2020	Cross-sectional	Amhara	6.0	4.0–22.0	8.5
Adem M, et al. [36]	2017	Cross-sectional	Oromia	2.19	1.12–4.28	7.5
Tesfe D, et al. [38]	2022	Cross-sectional	Amhara	6.33	3.34–11.99	9
Adem M, et al. [44]	2021	Cross-sectional	Amhara	0.997	0.594–1.72	8

**Notes:—CI-**Confidence interval.

**Table 5:** Study characteristics for patients’ co-morbid hypertension among diabetic patients in Ethiopia.

Authors	year	study design	Region	Odds ratio	95% CI	quality score
Aberra T, et al. [42]	2022	Cross-sectional	Addis Ababa	1.37	0.865–2.169	8.5
Adem M, et al. [44]	2021	Cross-sectional	Amhara	2.279	1.025–5.067	8
Alemu H, et al. [15]	2020	Cross-sectional	Amhara	8.2	2.0–23	8.5
Bekele MM, et al. [40]	2016	Cross-sectional	Addis Ababa	1.16	0.81–3.26	7
Damtie S, et al. [31]	2018	Cross-sectional	Amhara	4.51	2.266–8.977	9
Dinku B, et al. [33]	2022	Cross-sectional	Oromia	4.89	1.93–12.4	8.5
Taderegew MM, et al. [41]	2020	Cross-sectional	Amhara	3.12	1.51–6.45	8
Zemichael TM, et al. [5]	2020	Case-control	Tigray	6.44	4.46–9.28	8
Adem M, et al. [36]	2017	Cross-sectional	Oromia	5.62	2.81–11.23	7.5
Tesfe D, et al. [38]	2022	Cross-sectional	Amhara	4.85	2.07–11.3	9
Nibret E, et al. [43]	2020	Cohort	SNNPR	12.5	8.33–25.0	8.5
Fiseha T, et al. [39]	2020	Cross-sectional	Amhara	3.37	1.45–7.86	8

**Notes:-CI-**Confidence interval; **SNNP**-Southern Nations, Nationalities and Peoples.

### Determinant factors of diabetic nephropathy

#### Age of diabetic patients

**Fig 2:**
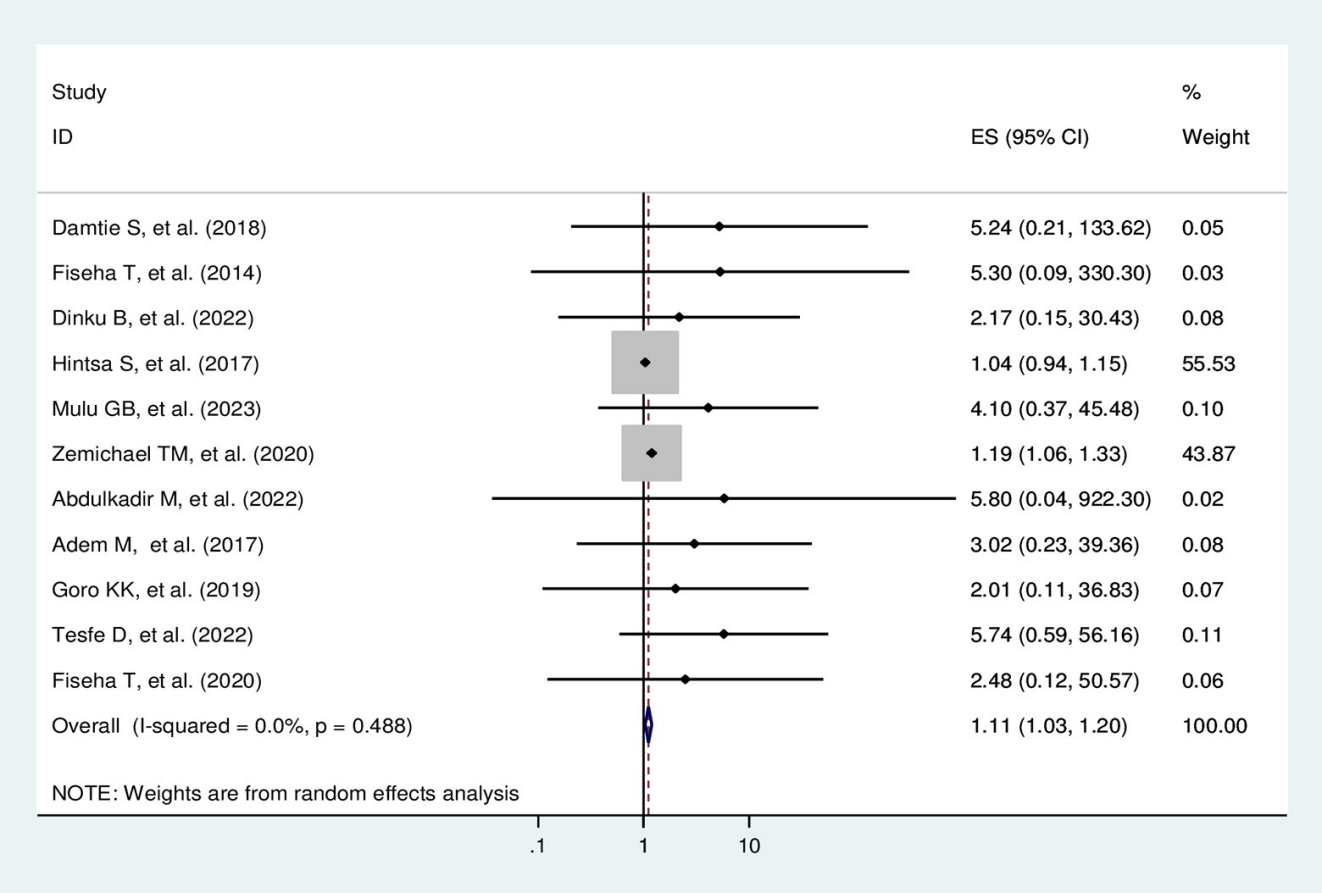
The pooled effect of age on diabetic nephropathy.

Diabetic patients with older age are 1.11 times more likely to develop DN compared to those diabetic patients with younger age (AOR = 1.11, 95% CI: 1.03–120, I^2^ = 0.0%, P = 0.488) (**Fig 2**).

#### Heterogeneity test and publication bias

**Fig 3:**
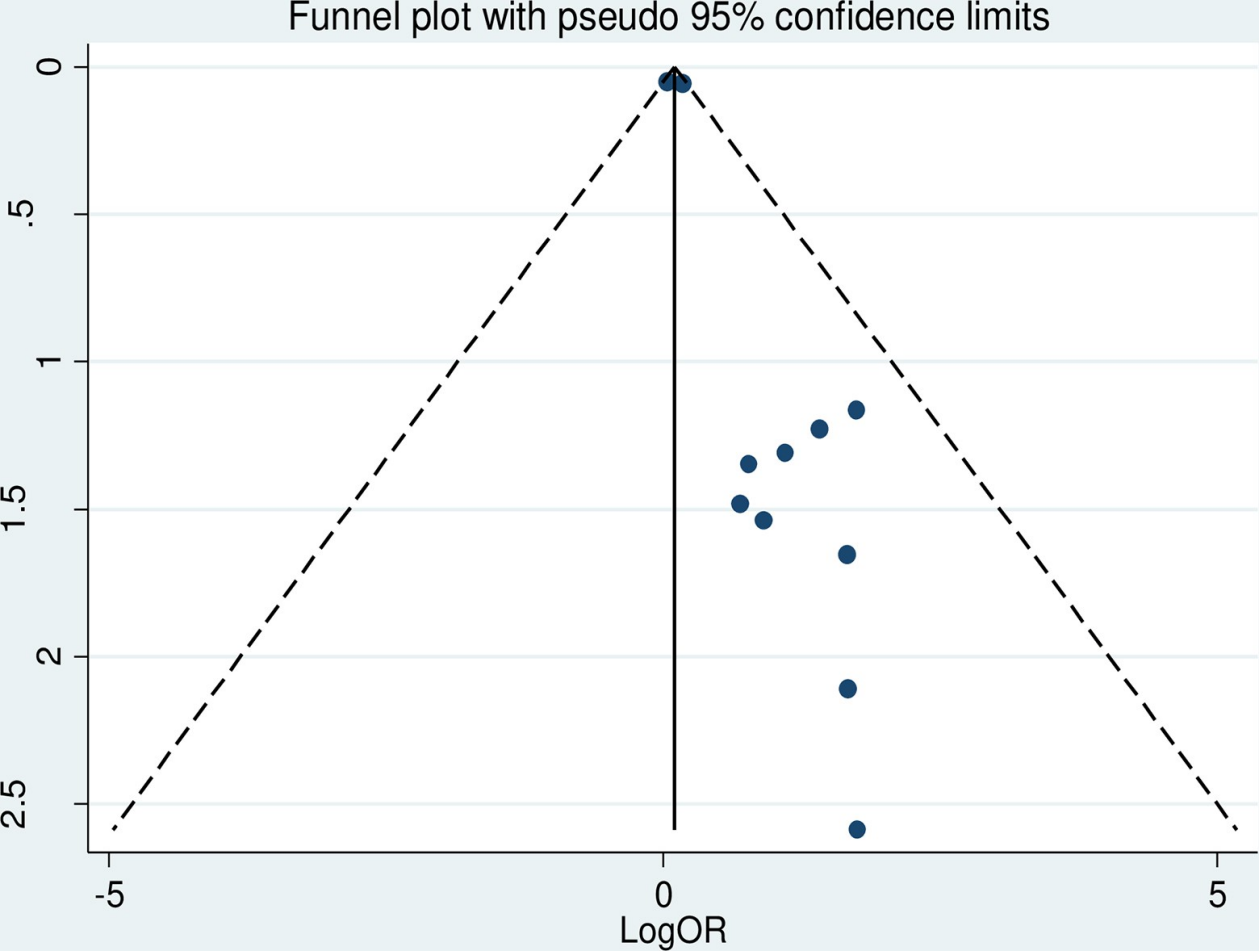
Funnel plot shows the distribution of included studies.

**Fig 4:**
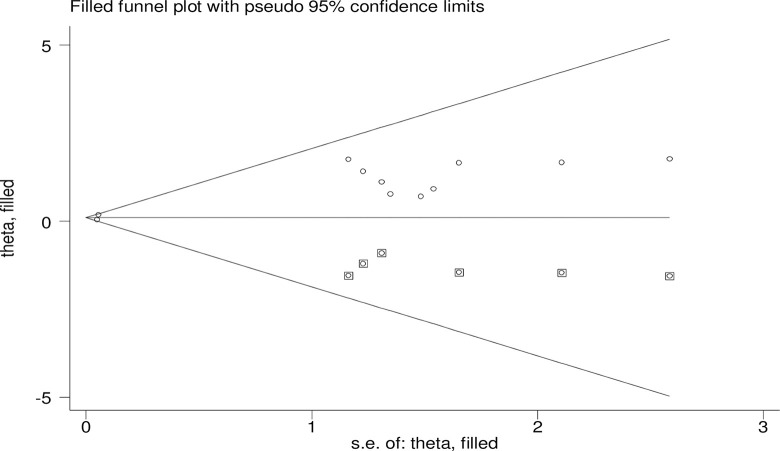
Trim and fill analysis for the asymmetrical distribution of the included studies.

The overall heterogeneity of the included study showed that I^2^ = 0.0% and the Cochrane Q statistic p-value = 0.488 using a random effect model, indicating there is no variation across studies. Regarding the publication bias, Egger’s test p-value is 0.005, and the funnel plot test result indicates asymmetrical (**Fig 3**) meaning that there is a publication bias. In order to treat the bias, a trim and fill analysis was carried out. Two iteration cycles were performed, and six articles were added, which made a total of 17 articles with 16 degrees of freedom, a p-value of 0.518, and a moment-based between study variance of 0.0 (**Fig 4**).

#### Duration of diabetic illness

**Fig 5:**
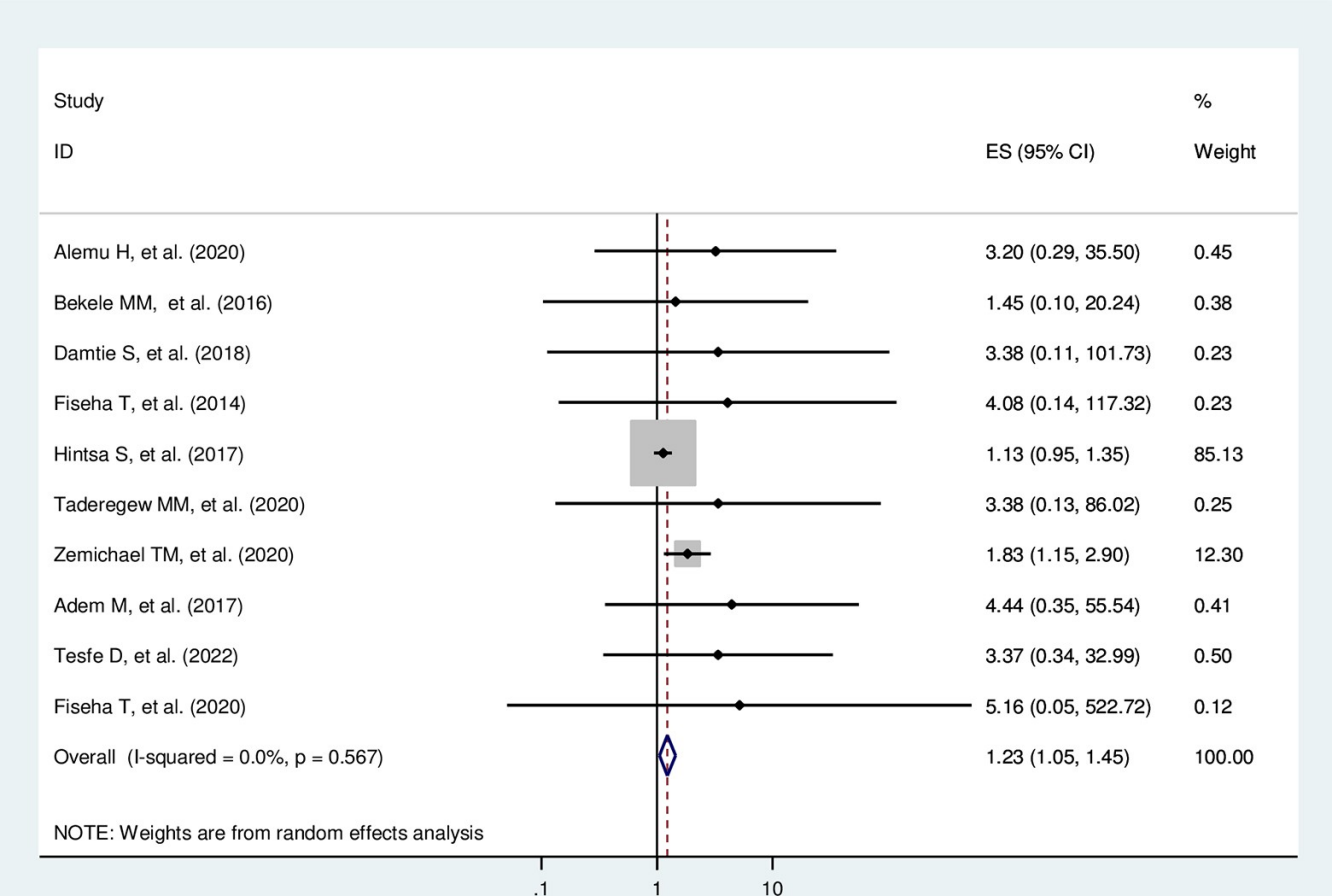
The pooled effect of duration of diabetes illness on Diabetic nephropathy.

Diabetic patients with a longer duration of diabetic illness are 1.23 times more likely to develop DN compared with diabetic patients with a shorter duration of diabetic illness (AOR = 1.23, 95% CI = 1.05–1.45, I^2^ = 0.0%, p = 0.567) (**Fig 5**).

#### Heterogeneity test and publication bias

**Fig 6:**
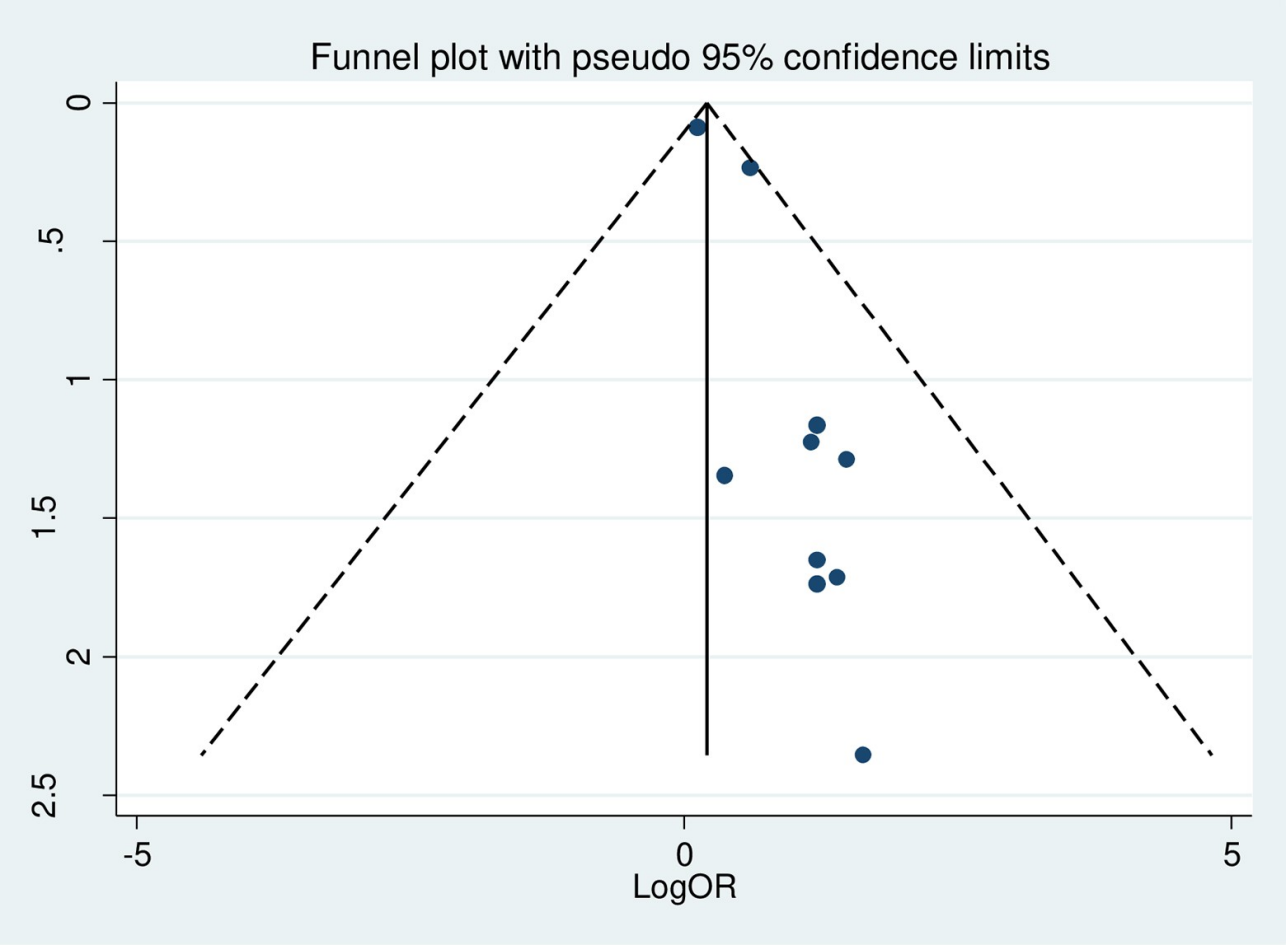
Funnel plot shows the distribution of the included studies.

**Fig 7:**
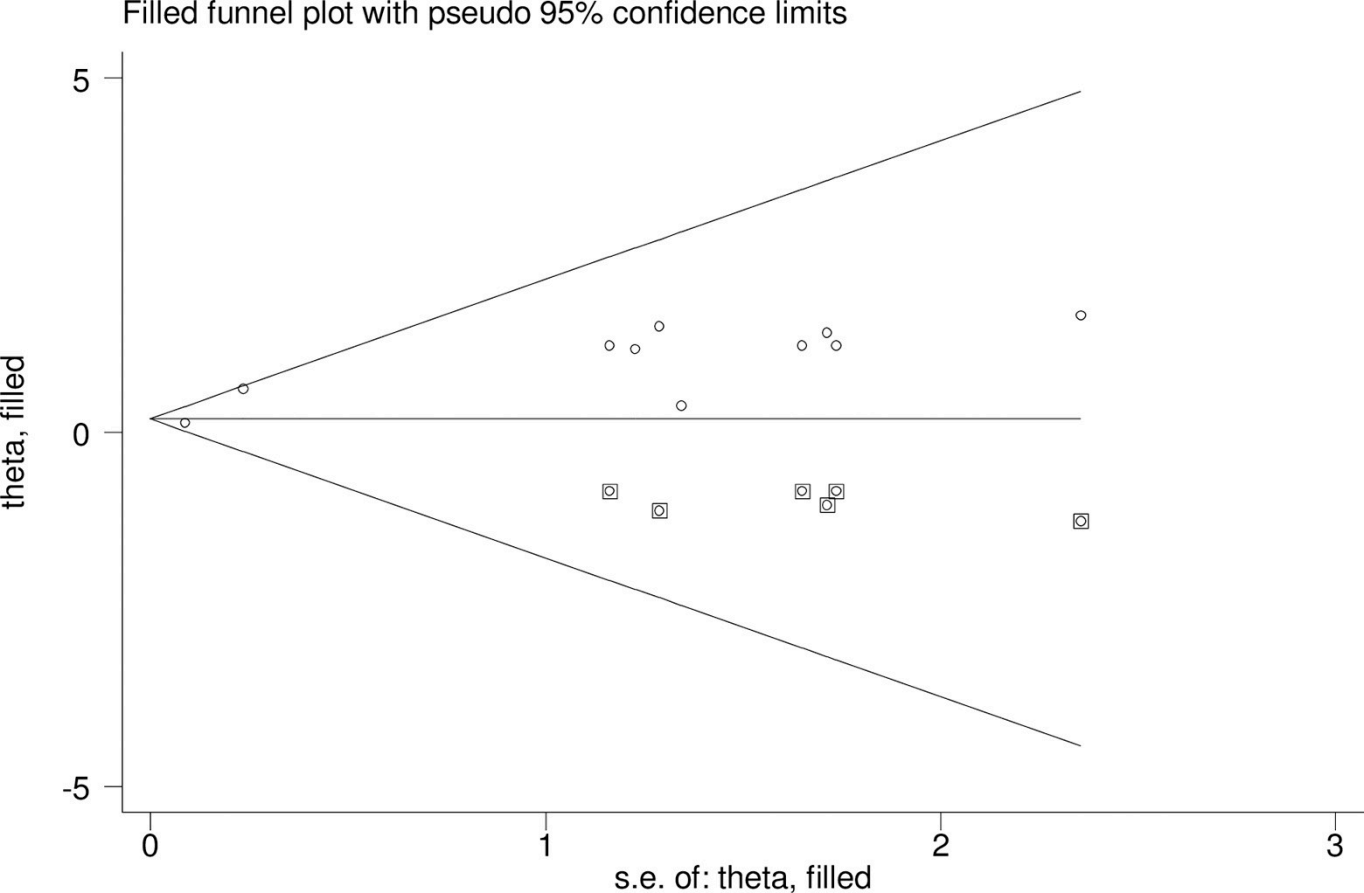
Trim and fill analysis for the asymmetrical distribution of the included studies.

The overall heterogeneity test of the included studies showed that I^2^ is nil and the Cochrane Q statistic p-value is 0.567 using a random effect model, indicating the included studies are homogenous. Concerning the publication bias, Egger’s test p-value is 0.004, and the graphic funnel plot test showed symmetrical (**Fig 6**), indicating there is publication bias across the studies. To treat the bias, trim and fill analysis was carried out, and two iteration cycles were performed, and six articles were added, making a total of 16 articles with 15 degrees of freedom, a p-value of 0.741, and a moment-based between study variance of 0.0 (**Fig 7**).

#### Poor glycemic control

**Fig 8:**
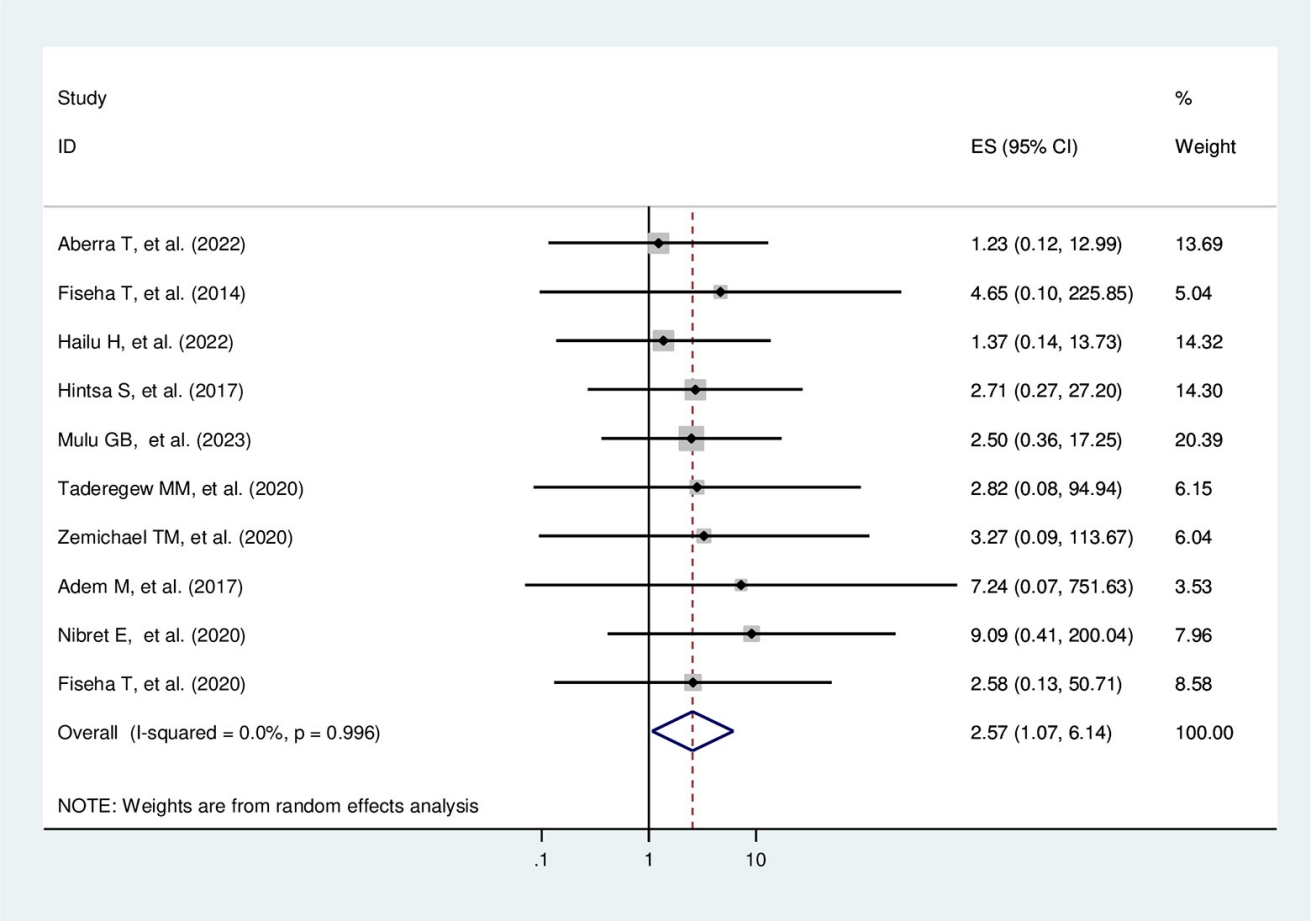
The pooled effect of poor glycemic control on diabetic nephropathy.

Diabetic patients who have poor glycemic control status are 2.57 times more likely to develop DN compared to those diabetic patients with good glycemic control status (AOR = 2.57, 95% CI: 1.07–6.14; I^2^ = 0.0%, p = 0.996) (**Fig 8**).

#### Heterogeneity test and publication bias

**Fig 9:**
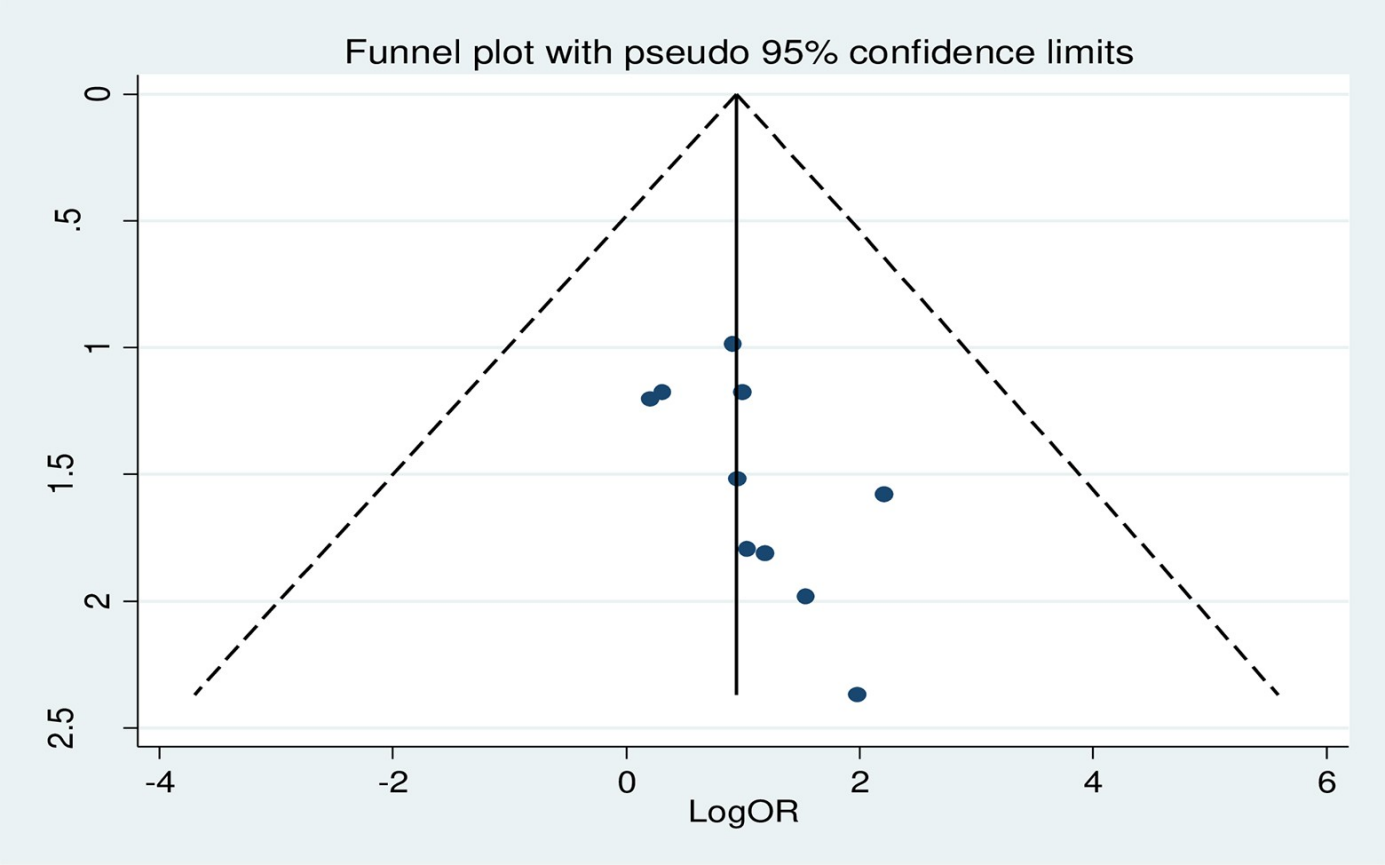
The funnel plot shows the distribution of the included studies.

The heterogeneity test of the studies indicates that I^2^ = 0.0% and the Cochrane Q statistic p-value is 0.996 using a random effect model, meaning that there is no variation across the included studies. The publication bias test result of Egger’s test p-value is 0.07, and the funnel plot test looks symmetrical (**Fig 9**), indicating there is no publication bias.

#### Elevated systolic hypertension

**Fig 10:**
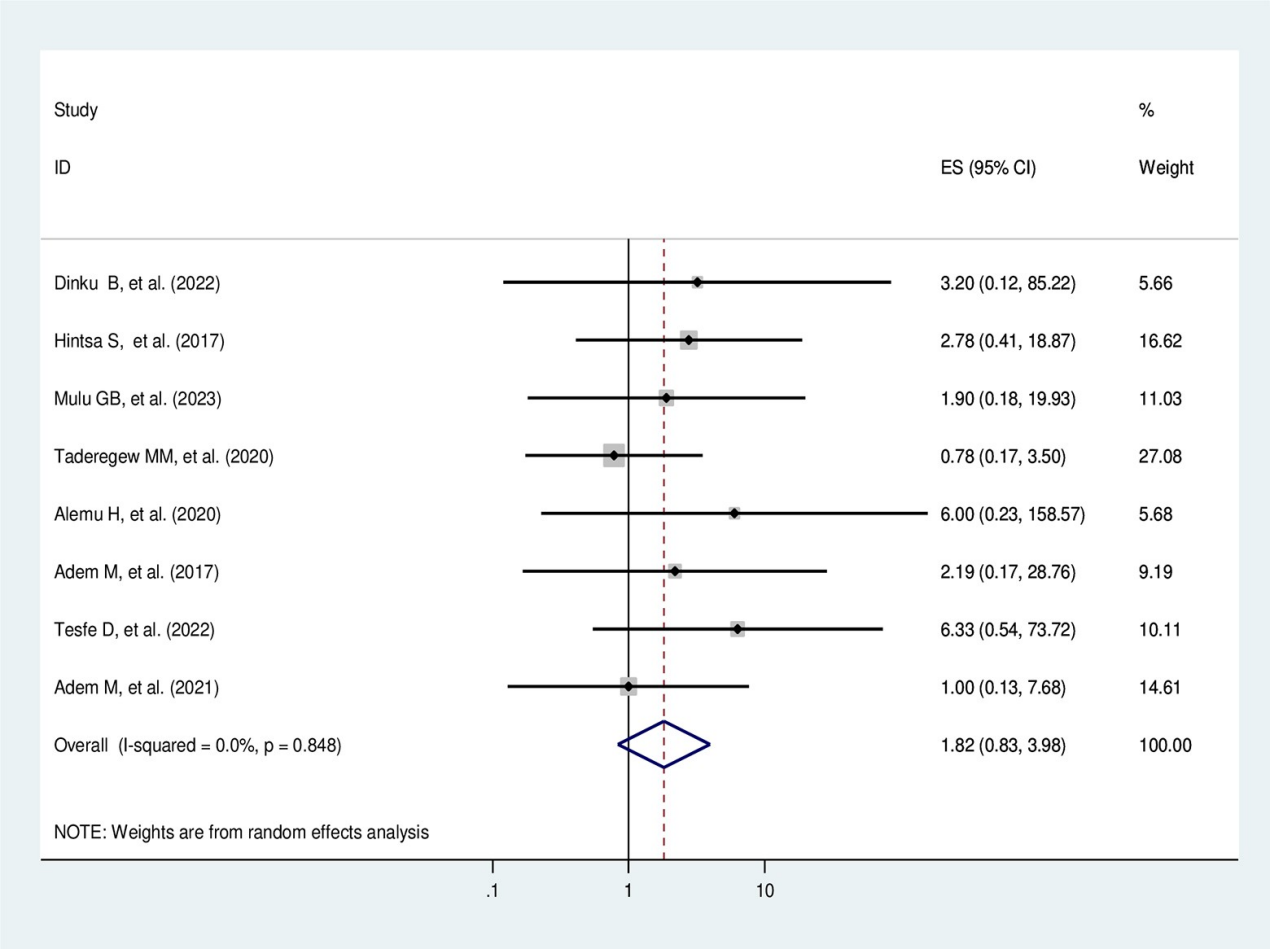
The pooled effect of elevated systolic hypertension on diabetic nephropathy.

Diabetic patients with elevated systolic hypertension is not at risk for developing DN (AOR = 1.82, 95%CI: 0.83–3.98; I^2^ = 0.0%, p = 0.848) **(Fig 10)**.

#### Co-morbid hypertension

**Fig 11:**
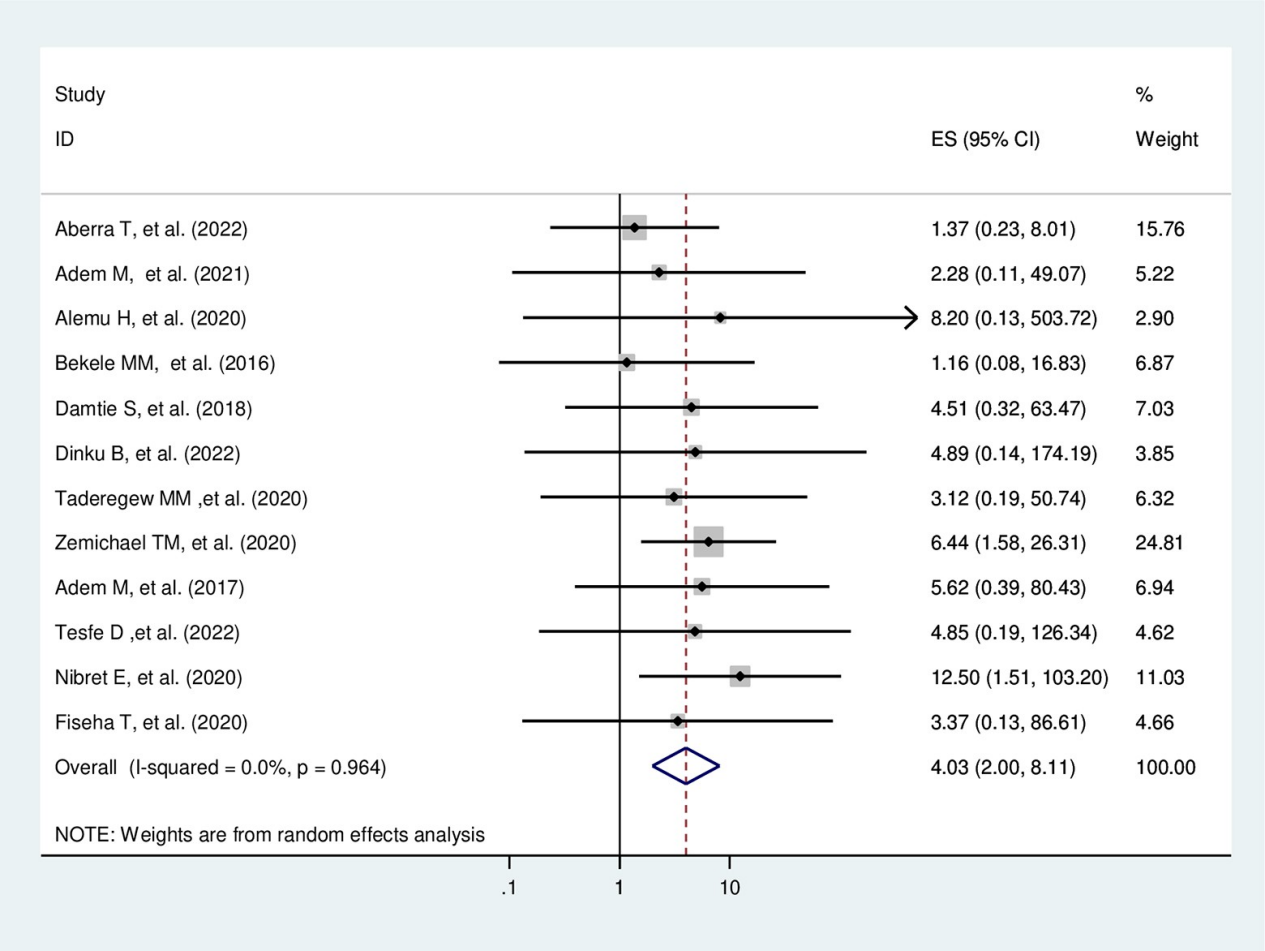
The pooled effect of co-morbid hypertension on diabetic nephropathy.

Diabetic patients with co-morbid hypertension are 4.03 times more likely to develop DN compared to diabetic patients without co-morbid hypertension (AOR = 4.03, 95%CI: 2.00–8.12, I^2^ = 0.0%, p = 0.964) (**Fig 11**).

#### Heterogeneity test and publication bias

**Fig 12:**
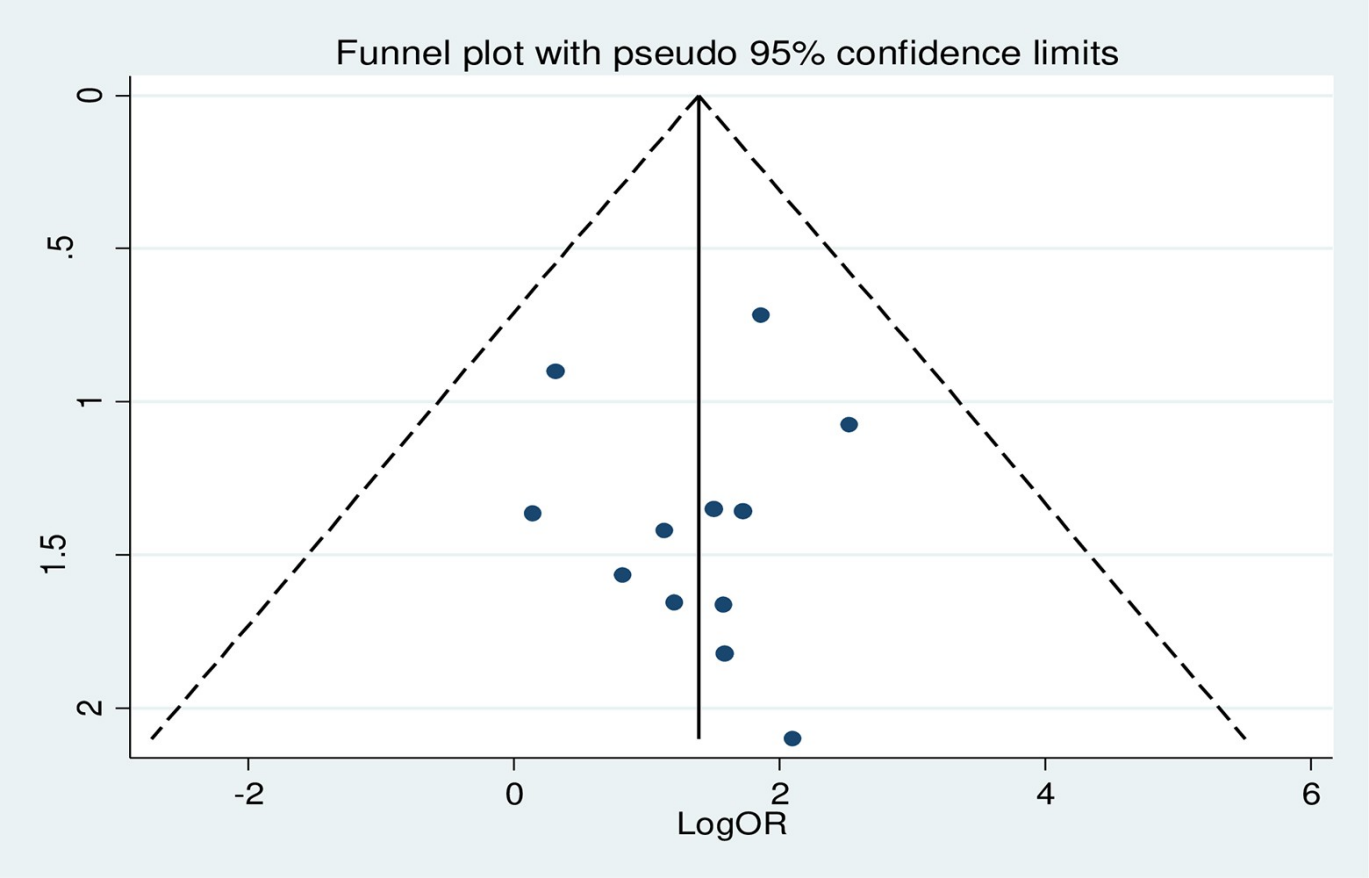
Funnel plot shows the distribution of the included studies.

As shown in the forest plot in **Fig 11** above, the I^2^ = 0.0% and the Cochrane Q statistic p-value is 0.964 using a random effect model, indicating there is no publication bias. Regarding the publication bias, the graphic asymmetry test of the funnel plot showed a symmetrical distribution (**Fig 12**), and the Eggers test p-value is 0.817, indicating there is no publication bias.

## Discussion

This systematic review and meta-analysis study identifies the important risk factors for DN. The findings of this study revealed that advanced age (≥55 years) is a risk factor for DN. The findings of the study is supported by the study conducted in Italy [45]. This is due to the fact that age plays a vital role in the deterioration of renal-vasculature functionality [46]. This problem worsens in the diabetic patient population [47]. This is due to advanced age, which results in stiffness of the blood vessels, eventually leading to renal vascular complications [48]. Furthermore, aging can predispose to various co-morbidities linked to several factors, such as oxidative stress, apoptosis, myocardial deterioration, and degeneration [49].

The duration of diabetic illness is the determinant factor for the development of DN. Patients with a longer duration of diabetic illness (≥10 years) are at risk of developing DN. This is consistent with the study conducted in the Netherlands [50]. This is due to the fact that uncontrolled blood glucose along with a longer duration of diabetic illness leads to renal vasculature damage, which impairs renal function [51].

The findings also depict that glycemic control status is the determinant factor of DN. Patients with poor glycemic control are at risk of developing DN. This is supported by the study in Iran [52]. Low socioeconomic status in medical care is responsible for poor glycemic control among diabetic patients [53]. Over time, poorly controlled diabetes can cause damage to blood vessel clusters in the kidneys that filter waste. High blood glucose affects the microvasculature of the kidneys, which results in nephrosclerosis [54].

Furthermore, co-morbid hypertension is found to be a risk factor for DN. The result of this study is supported by the studies conducted in Sub-Saharan Africa [55] and Australia [56]. This is because in Sub-Saharan studies, the similarity is due to poor lifestyle intervention and low socioeconomic status to prevent the occurrence of co-morbid hypertension [57]. The relationship between co-morbid hypertension and DN is not well investigated, but it is associated with renal artery sclerosis, and metabolic abnormalities derived from diabetes eventually lead to renal function deterioration [58]. Elevated hypertension is directly transmitted to the glomeruli. This leads to glomerular hypertension and the activation of mediators that induce inflammation, fibrosis, and further injury of the glomeruli [59]. High blood pressure causes further kidney damage by increasing the pressure in the delicate system of the kidney [60].

As a strong point, the authors used some of the important major databases to search for original research articles. In addition, the authors tried to incorporate different factors to estimate their effect on DN. On the contrary, the findings of this study have important limitations. 1) The study only contains observational study designs; 2) restricted only to the English language; 3) focused on the determinant factors of DN but not its prevalence; 4) the authors include both type 1 and/or type 2 diabetic patients; and 5) it incorporates specific factors. Therefore, the authors recommended further research that includes other than observational study designs; conduct on type 1 and type 2 diabetes patients separately; and other predictors for DN that need to be studied.

### Implications of the study

Nowadays, diabetic kidney disease is the main public health concern. As a result, the findings of this study provide input for policymakers to design effective diabetic care and complication prevention strategies. It also helps healthcare workers implement evidence-based interventions for diabetic patients with advanced age, living with a longer duration of diabetic illness, having poor glycemic control status, and having co-morbid hypertension to tackle the occurrence, burden, and severity of DN.

## Conclusions

The findings of this systematic review and meta-analysis study revealed that diabetic patients with advanced age, longer duration of diabetic illness, poor glycemic control status, and having co-morbid hypertension were found to be the determinant factors for DN. Therefore, treatment of co-morbid hypertension and blood glucose and regular screening of renal function should be implemented to reduce the occurrence of DN. Furthermore, healthcare workers should give due attention to diabetic patients with advanced age and a longer duration of diabetic illness to prevent the occurrence of DN.

## Supporting information

S1 File PRISMA checklist. (DOCX)

S2 File Data availability statement. (DOCX)

## Abbreviations

AOR: Adjusted Odds Ratio
CI: Confidence Interval
Co-HTN: co-morbid Hypertension
DN: Diabetic Nephropathy
DKD: diabetic Kidney disease
GFR: Glomerular filtration rate
OR: Odds Ratio
PRISMA: Preferred Reporting Item of Systematic Review and Meta-Analysis

## References

[1] Sun H SP, Karuranga S, Pinkepank M, Ogurtsova K, Duncan BB, et al. IDF Diabetes Atlas: Global, regional and country-level diabetes prevalence estimates for 2021 and projections for 2045. Diabetes Res Clin Pract. 2022;183. doi: 10.1016/j.diabres.2021.109119 34879977 PMC11057359

[2] Bishu KG JC, Yebyo HG, Atsbha M, Wubayehu T, Gebregziabher M. Diabetes in Ethiopia: A systematic review of prevalence, risk factors, complications, and cost. Obesity Medicine. 2019;15.

[3] Papatheodorou K PN, Banach M, Papazoglou D, Edmonds M. Complications of Diabetes 2016. J Diabetes Res 2016. 10.1155/2016/6989453 PMC5086373 27822482

[4] Lotfy M AJ, Kalasz H, Singh J, Adeghate E. Chronicnomplications of diabetes mellitus: a mini review. Curr Diabetes Rev. 2017;13(1):3–10. 26472574 10.2174/1573399812666151016101622

[5] Mariye Zemicheal T, Bahrey Tadesse D, Tasew Atalay H, Teklay Weldesamuel G, Gebremichael GB, Tesfay HN, et al. Determinants of Diabetic Nephropathy among Diabetic Patients in General Public Hospitals of Tigray, Ethiopia, 2018/19. International Journal of Endocrinology. 2020. doi: 10.1155/2020/6396483 33014045 PMC7525305

[6] Hussain S, Chand Jamali M, Habib A, Hussain MS, Akhtar M, Najmi AK. Diabetic kidney disease: An overview of prevalence, risk factors, and biomarkers. Clinical Epidemiology and Global Health. 2021;9:2–6.

[7] Parchwani D, Patel D. Incidence and Determinants of Incipient Diabetic Nephropathy. People’s Journal of Scientific Research. 2014;7(1):1–6.

[8] Koye DN MD, Nelson RG, Pavkov ME. The global epidemiology of diabetes and kidney disease. Advances in chronic kidney disease. 2018;25(2):121–32. doi: 10.1053/j.ackd.2017.10.011 29580576 PMC11000253

[9] Hintsa S, Dube L, Abay M, Angesom T, Workicho A. Determinants of diabetic nephropathy in Ayder Referral Hospital, Northern Ethiopia: a case-control study. PloS one. 2017;12(4):e0173566. doi: 10.1371/journal.pone.0173566 28403160 PMC5389619

[10] Kebede SA, Tusa BS, Weldesenbet AB, Tessema ZT, Ayele TA. Incidence of diabetic nephropathy and its predictors among type 2 diabetes mellitus patients at university of gondar comprehensive specialized hospital, Northwest Ethiopia. Journal of Nutrition and Metabolism. 2021;2021. doi: 10.1155/2021/6757916 34497725 PMC8419489

[11] Ritz E, Rychlík I, Locatelli F, Halimi S. End-stage renal failure in type 2 diabetes: a medical catastrophe of worldwide dimensions. American journal of kidney diseases. 1999;34(5):795–808. doi: 10.1016/S0272-6386(99)70035-1 10561134

[12] Zhang X-X, Kong J, Yun K. Prevalence of diabetic nephropathy among patients with type 2 diabetes mellitus in China: a meta-analysis of observational studies. Journal of diabetes research. 2020;2020. 10.1155/2020/2315607 PMC7023800 32090116

[13] Wu A, Kong N, De Leon F, Pan C, Tai T, Yeung V, et al. An alarmingly high prevalence of diabetic nephropathy in Asian type 2 diabetic patients: the MicroAlbuminuria Prevalence (MAP) Study. Diabetologia. 2005;48:17–26. doi: 10.1007/s00125-004-1599-9 15616801

[14] Al-Rubeaan K, Youssef AM, Subhani SN, Ahmad NA, Al-Sharqawi AH, Al-Mutlaq HM, et al. Diabetic nephropathy and its risk factors in a society with a type 2 diabetes epidemic: a Saudi National Diabetes Registry-based study. PloS one. 2014;9(2):e88956. doi: 10.1371/journal.pone.0088956 24586457 PMC3931705

[15] Alemu H, Hailu W, Adane A. Prevalence of chronic kidney disease and associated factors among patients with diabetes in northwest Ethiopia: a hospital-based cross-sectional study. Current Therapeutic Research. 2020;92:100578. doi: 10.1016/j.curtheres.2020.100578 32190131 PMC7068620

[16] Tuttle KR BG, Bilous RW, Chiang JL, et al. Diabetic kidney disease: a report from an ADA Consensus Conference. Diabetes Care. 2014;37(10):2864–83. doi: 10.2337/dc14-1296 25249672 PMC4170131

[17] Williams R, Karuranga S, Malanda B, Saeedi P, Basit A, Besançon S, et al. Global and regional estimates and projections of diabetes-related health expenditure: Results from the International Diabetes Federation Diabetes Atlas. Diabetes research and clinical practice. 2020;162:108072. 32061820 10.1016/j.diabres.2020.108072

[18] Gregg EW, Buckley J, Ali MK, Davies J, Flood D, Mehta R, et al. Improving health outcomes of people with diabetes: target setting for the WHO Global Diabetes Compact. The Lancet. 2023;401(10384):1302–12. doi: 10.1016/S0140-6736(23)00001-6 36931289 PMC10420388

[19] Webster AC, Nagler EV, Morton RL, Masson P. Chronic kidney disease. The lancet. 2017;389(10075):1238–52. 10.1016/S0140-6736(16)32064-5 27887750

[20] Page MJ, McKenzie JE, Bossuyt PM, Boutron I, Hoffmann TC, Mulrow CD, et al. The PRISMA 2020 statement: an updated guideline for reporting systematic reviews. International journal of surgery. 2021;88:105906. doi: 10.1016/j.ijsu.2021.105906 33789826

[21] Moskalewicz A, Oremus M. No clear choice between Newcastle–Ottawa Scale and Appraisal Tool for Cross-Sectional Studies to assess methodological quality in cross-sectional studies of health-related quality of life and breast cancer. Journal of clinical epidemiology. 2020;120:94–103. 31866469 10.1016/j.jclinepi.2019.12.013

[22] Wells G, Shea B, O’Connell D, Peterson J, Welch V, Losos M, et al. Newcastle-Ottawa quality assessment scale cohort studies. University of Ottawa. 2014.

[23] Wells G, Shea B, O’connell D, Peterson J, Welch V, Losos M, et al. NewCastle–Ottawa quality assessment scale—case control studies. Belia Vida Centre, Namibia. 2017.

[24] McPheeters ML, Kripalani S, Peterson NB, Idowu RT, Jerome RN, Potter SA, et al. Quality improvement interventions to address health disparities: closing the quality gap-revisiting the state of the science. Database of Abstracts of Reviews of Effects (DARE): Quality-assessed Reviews [Internet]. 2012. PMC4781280 24422952

[25] Tuttle KR BG, Bilous RW, Chiang JL, et al. Diabetic kidney disease: a report from an ADA Consensus Conference. Diabetes Care. 2014;37(10):2864–83. doi: 10.2337/dc14-1296 25249672 PMC4170131

[26] Rücker G, Schwarzer G, Carpenter JR, Schumacher M. Undue reliance on I 2 in assessing heterogeneity may mislead. BMC medical research methodology. 2008;8:1–9. 19036172 10.1186/1471-2288-8-79 PMC2648991

[27] Borenstein M, Hedges LV, Higgins JP, Rothstein HR. A basic introduction to fixed‐effect and random‐effects models for meta‐analysis. Research synthesis methods. 2010;1(2):97–111. doi: 10.1002/jrsm.12 26061376

[28] Song F, Khan KS, Dinnes J, Sutton AJ. Asymmetric funnel plots and publication bias in meta-analyses of diagnostic accuracy. International journal of epidemiology. 2002;31(1):88–95. doi: 10.1093/ije/31.1.88 11914301

[29] Begg CB, Mazumdar M. Operating characteristics of a rank correlation test for publication bias. Biometrics. 1994:1088–101. 7786990

[30] Duval S, Tweedie R. Trim and fill: a simple funnel‐plot–based method of testing and adjusting for publication bias in meta‐analysis. Biometrics. 2000;56(2):455–63. doi: 10.1111/j.0006-341x.2000.00455.x 10877304

[31] Damtie S, Biadgo B, Baynes HW, Ambachew S, Melak T, Asmelash D, et al. Chronic kidney disease and associated risk factors assessment among diabetes mellitus patients at a tertiary hospital, Northwest Ethiopia. Ethiopian journal of health sciences. 2018;28(6). 10.4314/ejhs.v28i6.3 PMC6308752 30607085

[32] Fiseha T, Kassim M, Yemane T. Prevalence of chronic kidney disease and associated risk factors among diabetic patients in southern Ethiopia. Am J Health Res. 2014;2(4):216–21.

[33] Hailu HE, Dinku B, Wondimu J, Girma B. Prevalence and Associated Factors of Chronic Kidney Disease Among Diabetic and Hypertensive Patients at Ambo Town Public Hospitals of West Shewa Zone, Oromia Region, Ethiopia. 2022.

[34] Mulu GB, Kebede WM, Tarekegn FN, Engida AS, Tiruye ME, Menalu MM, et al. The prevalence and risk factors of chronic kidney disease among type 2 diabetes mellitus follow-up patients at Debre Berhan Referral Hospital, Central Ethiopia. 2023.

[35] Abdulkadr M, Merga H, Mizana BA, Terefe G, Dube L. Chronic Kidney Disease and Associated Factors among Diabetic Patients at the Diabetic Clinic in a Police Hospital, Addis Ababa. Ethiopian Journal of Health Sciences. 2022;32(2). 10.4314/ejhs.v32i2.11 PMC9175221 35693577

[36] Mohammed Adem AG, Shiferaw Bekele(MSc). Renal impairrment and associated factors among type 1 and type 2 diabetic patients at Jimma University medical center, South west Ethiopia 2017.

[37] Kumela Goro K, Desalegn Wolide A, Kerga Dibaba F, Gashe Fufa F, Wakjira Garedow A, Edilu Tufa B, et al. Patient awareness, prevalence, and risk factors of chronic kidney disease among diabetes mellitus and hypertensive patients at Jimma University Medical Center, Ethiopia. BioMed Research International. 2019;2019. doi: 10.1155/2019/2383508 31214611 PMC6535886

[38] Tesfe D, Adugna M, Nigussie ZM, Woldeyohanins AE, Kifle ZD. The proportion of chronic kidney disease and its associated factors among adult diabetic patients at Tibebe Ghion Specialized Hospital, Bahir Dar, Ethiopia. Metabolism Open. 2022;15:100198. doi: 10.1016/j.metop.2022.100198 35785137 PMC9241132

[39] Fiseha T, Tamir Z. Prevalence and awareness of chronic kidney disease among adult diabetic outpatients in Northeast Ethiopia. BMC nephrology. 2020;21(1):1–7. doi: 10.1186/s12882-020-01768-y 32293323 PMC7161144

[40] Meron Moges Bekele ND, Bisrat Alem. Prevalence and Associated Factors of Chronic Kidney Disease among Diabetic Patients that attend Public Hospitals of Addis Ababa. 2016.

[41] Taderegew MM. Assessment of renal impairment using estimated glomerular filtration rate among type 2 diabetes mellitus patients in North-East Ethiopia: a cross-sectional study. Journal of Diabetes & Metabolic Disorders. 2020;19:1473–81. doi: 10.1007/s40200-020-00680-4 33520848 PMC7843698

[42] Aberra T, Feleke Y, Tarekegn G, Bikila D, Melesse M. Prevalence and associated factors of diabetic nephropathy at Tikur Anbessa Comprehensive Specialized University Hospital, Addis Ababa, Ethiopia. African Journal of Nephrology. 2022;25(1):35–45.

[43] Nibret E, Worku G, Zewdie A. The Prevalence of Chronic Kidney Disease and Associated Factors among Type-2 Diabetic Patients Admitted to Dilla University Referral Hospital Medical, Ward, Snnpr, Ethiopia, 2019. morbidity and mortality. 2020;10:11.

[44] Mohammed Adem WM. Assessment of The Prevalence and Associated Factors of Chronic Kidney Disease Among Diabetes Mellitus Patients at Dessie Referral Hospital, South Wollo, Ethiopia 2021. 10.1038/s41598-024-59184-3 PMC11035645 38649429

[45] Russo GT, De Cosmo S, Viazzi F, Mirijello A, Ceriello A, Guida P, et al. Diabetic kidney disease in the elderly: prevalence and clinical correlates. BMC geriatrics. 2018;18(1):1–11. 29394888 10.1186/s12877-018-0732-4 PMC5797340

[46] North BJ, Sinclair DA. The intersection between aging and cardiovascular disease. Circulation research. 2012;110(8):1097–108. doi: 10.1161/CIRCRESAHA.111.246876 22499900 PMC3366686

[47] Rodgers JL, Jones J, Bolleddu SI, Vanthenapalli S, Rodgers LE, Shah K, et al. Cardiovascular risks associated with gender and aging. Journal of cardiovascular development and disease. 2019;6(2):19. doi: 10.3390/jcdd6020019 31035613 PMC6616540

[48] Abdelhafiz AH. Diabetic kidney disease in older people with type 2 diabetes mellitus: improving prevention and treatment options. Drugs & Aging. 2020;37:567–84. 10.1007/s40266-020-00773-y 32495289

[49] Divo MJ, Martinez CH, Mannino DM. Ageing and the epidemiology of multimorbidity. European Respiratory Journal. 2014;44(4):1055–68. doi: 10.1183/09031936.00059814 25142482 PMC4918092

[50] Vinter-Repalust N, Jurković L, Katić M, Simunović R, Petric D. Disease duration, patient compliance and presence of complications in diabetic patients. Acta Medica Croatica: Casopis Hravatske Akademije Medicinskih Znanosti. 2007;61(1):57–62. 17593642

[51] Zoungas S, Woodward M, Li Q, Cooper ME, Hamet P, Harrap S, et al. Impact of age, age at diagnosis and duration of diabetes on the risk of macrovascular and microvascular complications and death in type 2 diabetes. Diabetologia. 2014;57:2465–74. doi: 10.1007/s00125-014-3369-7 25226881

[52] Shahbazian H, Rezaii I. Diabetic kidney disease; review of the current knowledge. Journal of renal injury prevention. 2013;2(2):73. doi: 10.12861/jrip.2013.24 25340133 PMC4206005

[53] Zawudie AB, Daka DW, Teshome D, Ergiba MS. Economic burden of diabetic mellitus among patients on follow-up care in Hospitals of Southwest Shewa Zone, central Ethiopia. BMC Health Services Research. 2022;22(1):1–15. 36419111 10.1186/s12913-022-08819-0 PMC9685907

[54] Reutens AT, Atkins RC. Epidemiology of diabetic nephropathy. Diabetes and the Kidney. 2011;170:1–7. doi: 10.1159/000324934 21659752

[55] Wagnew F, Eshetie S, Kibret GD, Zegeye A, Dessie G, Mulugeta H, et al. Diabetic nephropathy and hypertension in diabetes patients of sub-Saharan countries: a systematic review and meta-analysis. BMC research notes. 2018;11:1–7. 30081966 10.1186/s13104-018-3670-5 PMC6080368

[56] Leutner M, Haug N, Bellach L, Dervic E, Kautzky A, Klimek P, et al. Risk of typical diabetes-associated complications in different clusters of diabetic patients: analysis of nine risk factors. Journal of Personalized Medicine. 2021;11(5):328. doi: 10.3390/jpm11050328 33922088 PMC8143487

[57] Stephani V, Opoku D, Beran D. Self-management of diabetes in Sub-Saharan Africa: a systematic review. BMC public health. 2018;18:1–11. doi: 10.1186/s12889-018-6050-0 30268115 PMC6162903

[58] Baba T, Neugebauer S, Watanabe T. Diabetic nephropathy: its relationship to hypertension and means of pharmacological intervention. Drugs. 1997;54(2):197–234. 9257079 10.2165/00003495-199754020-00002

[59] Van Buren PN, Toto R. Hypertension in diabetic nephropathy: epidemiology, mechanisms, and management. Advances in chronic kidney disease. 2011;18(1):28–41. 10.1053/j.ackd.2010.10.003 PMC3221014 21224028

[60] Stanton R. Diabetic kidney disease and hypertension. Experimental and Clinical Endocrinology & Diabetes. 2015:93–8. doi: 10.1055/s-0035-1559760 26575123

