# Supplementary material for: Determinants of diabetic nephropathy among diabetic patients in Ethiopia: Systematic review and meta-analysis
Source: PLoS One. 2024 Feb 2;19(2):e0297082. doi: 10.1371/journal.pone.0297082 (PMC10836702; doi:10.1371/journal.pone.0297082)
Supplement: S1 File — (DOCX) [file pone.0297082.s001.docx]

| **Section and Topic** | **Item #** | **Checklist item** | **Location where item is reported** |
| --- | --- | --- | --- |
| **TITLE** | | |  |
| Title | 1 | **Determinants of diabetic nephropathy among diabetic patients in Ethiopia: Systematic review and meta-analysis** | 1 |
| **ABSTRACT** | | |  |
| Abstract | 2 | Diabetic nephropathy (DN) is the long-term kidney disease that can affect people with diabetes. It is the leading public health challenge globally which results in end stage renal failure. Nearly one fifth (18.22%) of diabetic population in Ethiopia affected with DN but there were inconsistent findings across studies about the determinant factors of DN. Therefore, the aim of this study is to identify the determinant factors of DN.  **Method:** We have accessed previous studies through electronic web-based search strategies using PubMed, OVID, Embsco, Embase, Google, and Google Scholar, with a combination of search terms by Boolean operators. The quality of each included article was assessed using the Newcastle Ottawa Assessment Scale. All statistical analyses were carried out using Stata version14 software. The odds ratios of risk factors were pooled using a random-effect meta-analysis model. Heterogeneity was assessed using the Cochrane Q statistics and I-Square (I^2^). Furthermore, publication bias was detected based on the graphic asymmetry test of the funnel plot and/or Egger’s test (p< 0.05). Furthermore, trim and fill analysis was carried out to treat the publication bias.  **Result:** The search strategy retrieved 1145 both published and unpublished original articles. After the removal of duplicate articles, 127 articles remained. Following further screening, about 19 articles were assessed for eligibility, of which three articles were excluded because of reporting without the outcome of interest, poor quality, and not full text. Finally, sixteen articles were reviewed for the final analysis. Diabetic patients with advanced age (AOR= 1.11, 95% CI: 1.03,120, I^2^= 0.0%, p= 0.488), longer duration of diabetic illness (AOR=1.23, 95% CI= 1.05, 1.45, I^2^= 0.0%, p=0.567), poor glycemic control (AOR=2.57, 95%CI: 1.07, 6.14; I^2^ = 0.0%, p=0.996), and having co-morbid hypertension (AOR=4.03, 95%CI: 2.00-8.12, I^2^ = 0.0%, p- 0.964) were found factors associated with diabetic nephropathy.  **Conclusion**: The finding of this systematic review and meta analysis study revealed that, diabetic patients with advanced age, longer duration of diabetic illness, poor glycemic control status, and having co-morbid hypertension were found the determinant factors of diabetic nephropathy. Therefore, Treatment of co-morbid hypertension and blood glucose, and regular screening of renal function should be implemented to reduce the occurrence of diabetic kidney diseases.  **Keywords:** Diabetic nephropathy, Diabetes mellitus, Determinant factors, Ethiopia | 2 |
| **INTRODUCTION** | | |  |
| Rationale | 3 | Diabetic nephropathy, or Diabetic Kidney Disease (DKD), is one of the late complications caused by uncontrolled diabetes mellitus, a significant public health problem. Usually characterized by increased urine albumin excretion (microalbuminuria), decreased glomerular filtration rate, or both. Like chronic kidney disease (CKD) in diabetic patients, DKD is increasing significantly in low and middle-income countries while remaining under-recognized as a global illness burden. Even though various studies have been undertaken on the severity of diabetic nephropathy, the knowledge of the factors of diabetic nephropathy is inconsistent across different studies among diabetes mellitus patients worldwide, with Ethiopia being no exception. Early detection of determinants is the mainstay of early screening and therapy, decreasing disease progression and its associated consequences. As a result, the purpose of this review aimed to investigate the determinants for diabetic nephropathy in people with diabetes. | 3 |
| Objectives | 4 | To identify factors associated with diabetic nephropathy among diabetic patients in Ethiopia |  |
| **METHODS** | | |  |
| Eligibility criteria | 5 | In this systematic review and meta-analysis study, articles which fulfill the eligibility criteria: (1) articles with observational studies such as cross-sectional, cohort, and case control; (2) articles that report the determinant /risk factors/ associated factors of diabetic nephropathy; (3) studies conducted in Ethiopia; (4) both published and/or unpublished articles; (5) articles published/conducted at any time; (6) articles written by English; (7) articles conducted at community or health facility settings were included in the study. Articles with trials, without full text, conference papers, and systematic review and meta-analysis were excluded in the study. | 6 |
| Information sources | 6 | We search on electronic databases such as PubMed, Embase, EBSECO, Web of Sciences, OVID, and search engines (Google and Google Scholars) for grey literatures. | 4 |
| Search strategy | 7 | The search strategies were carried out using controlled vocabularies (MeSH terms). The synonym of diabetic nephropathy was identified. Then, the search string was established using the databases. Articles were searched by title (ti), abstract (ab), and/or full-text (ft). Boolean logic operators; “AND” and “OR” were used to combine the search terms. | 4 |
| Selection process | 8 | Eligible research articles were screened by their title (Ti), Abstract (Ab), and full-text. Two reviewers independently reviewed the included articles. | 4 |
| Data collection process | 9 | After the quality assessment of the studies, the data were extracted using the Microsoft excel speed sheet. The extracted data item includes; authors, publication year, study design, region of the study, data collection method, and funding source. Two authors/reviewers were independently extracted the data. The disagreements between the reviewers were resolved by discussion and third reviewer involvement. | 6 |
| Data items | 10a | Articles that have clearly defined effect meaures were included | 6 |
|  | 10b | The variables that have direct effect for the occurrence of Diabetic nephrophathy were identified. | 6 |
| Study risk of bias assessment | 11 | The quality of the study was evaluated using the Newcastle Ottawa quality assessment tool adapted from the cross-sectional, cohort, and case-control studies. To reviewers were independently reviewed the quality of the included studies. The inconveniences between the reviewers were resolved by discussion. A score of ≥7 was considered as high quality score. | 6 |
| Effect measures | 12 | The odds ratio (or), logor, and standard error or (SeOr) were used presentation of results. | 6 |
| Synthesis methods | 13a | Both qualitative synthesis and quantitative analysis were employed. | 4 |
|  | 13b | The crud odds ratio(COR) or adjusted odds ratio(AOR) were used for data presentation or evidence synthesis. | 4 |
|  | 13c | PRISMA flow chart, forest plot, and funnel plot were used to present visually displayed data. | 5 |
|  | 13d | The Cochrane Q statistics and (**I**^2^) were used to assess the heterogeneity status of the included studies. | 7 |
|  | 13e | A fixed effect model was used for analysis. | 7 |
|  | 13f | The quantitative synthesis was employed | 7 |
| Reporting bias assessment | 14 | Begg’s and/or Egger’s test was computed to detect publication bias. | 7 |
| Certainty assessment | 15 | The pooled summary effect size of the study was estimated | 7 |
| **RESULTS** | | |  |
| Study selection | 16a | The articles were screened by title, abstract, and full text. Two independent reviewers screened the studies, and any controversy was resolved by discussion. | 8 |
|  | 16b | Studies were excluded because of outcome interest and not full text articles. | 7 |
| Study characteristics | 17 | Of these, eleven articles were retrieved for age of diabetic patients [[9](#_ENREF_9), [18](#_ENREF_18), [33-41](#_ENREF_33)] (**Table 1**), ten for duration of diabetic illness[[9](#_ENREF_9), [12](#_ENREF_12), [18](#_ENREF_18), [33](#_ENREF_33), [34](#_ENREF_34), [38](#_ENREF_38), [40-43](#_ENREF_40)] **(Table 2)**, ten for poor glyceic control[[9](#_ENREF_9), [16](#_ENREF_16), [18](#_ENREF_18), [34-36](#_ENREF_34), [38](#_ENREF_38), [41](#_ENREF_41), [43](#_ENREF_43), [44](#_ENREF_44)] (**Table 3)**, eight for elevated systolic blood pressure [[12](#_ENREF_12), [18](#_ENREF_18), [35](#_ENREF_35), [36](#_ENREF_36), [38](#_ENREF_38), [40](#_ENREF_40), [43](#_ENREF_43), [45](#_ENREF_45)] **(Table 4)**, and twelve for co-morbid hypertension [[9](#_ENREF_9), [12](#_ENREF_12), [16](#_ENREF_16), [33](#_ENREF_33), [35](#_ENREF_35), [38](#_ENREF_38), [40-45](#_ENREF_40)] (**Table 5)**. From the included studies seven in the Amhara [[12](#_ENREF_12), [33](#_ENREF_33), [36](#_ENREF_36), [40](#_ENREF_40), [41](#_ENREF_41), [43](#_ENREF_43), [45](#_ENREF_45)], four in Oromia [[35](#_ENREF_35), [38](#_ENREF_38), [39](#_ENREF_39)], two in Addis Ababa [[16](#_ENREF_16), [42](#_ENREF_42)], two in Tgray [[9](#_ENREF_9), [18](#_ENREF_18)] and two in SNNP [[34](#_ENREF_34), [44](#_ENREF_44)] regions of Ethiopia. Regarding the study design, as shown in each table, most of the studies were cross-sectional studies. Majority of the studies used both patient interview and record review for data collection. Most of the included studies were not funded and/their funding status were unreported. The average quality appraisal score of the included studies was ≥7. . | 7 |
| Risk of bias in studies | 18 | In terms of their quality status, all the included studies have high quality | 8 |
| Results of individual studies | 19 | \| **Authors** \| **year** \| **study**  **design** \| **Region** \| **data collection** \| **Funding source** \| **Factors** \| **Odds ratio** \| **95%**  **CI** \| **quality score** \| \| --- \| --- \| --- \| --- \| --- \| --- \| --- \| --- \| --- \| --- \| \| Damtie S, et al.[[31](#_ENREF_31)] \| 2018 \| Cross Sectional \| Amhara \| patient interview, record review \| Not funded \| age \| 5.239 \| 2.255-12.175 \| 9 \| \| Fiseha T, et al.[[32](#_ENREF_32)] \| 2014 \| Cross Sectional \| SNNP \| patient interview \| Not reported \| age \| 5.3 \| 1.81-15.56 \| 8 \| \| Dinku B, et al.[[33](#_ENREF_33)] \| 2022 \| Cross Sectional \| Oromia \| patient interview, record review \| St PMMC \| age \| 2.17 \| 1.09-4.31 \| 8.5 \| \| Hintsa S, et al.[[9](#_ENREF_9)] \| 2017 \| Case control \| Tigray \| record review \| Not funded \| age \| 1.037 \| 1.01-1.064 \| 7 \| \| Mulu GB, et al.[[34](#_ENREF_34)] \| 2023 \| Cross Sectional \| Amhara \| patient interview \| Not reported \| age \| 4.1 \| 2.2-7.7 \| 7.5 \| \| Zemichael TM, et al.[[5](#_ENREF_5)] \| 2020 \| Case control \| Tigray \| patient interview \| Not reported \| age \| 1.19 \| 1.16-1.23 \| 8 \| \| Abdulkadir M, et al.[[35](#_ENREF_35)] \| 2022 \| Cross Sectional \| Addis Ababa \| patient interview \| Not funded \| age \| 5.8 \| 1.5-21 \| 8 \| \| Adem M, et al.[[36](#_ENREF_36)] \| 2017 \| Cross Sectional \| Oromia \| patient interview \| Not funded \| age \| 3.02 \| 1.55-5.9 \| 7.5 \| \| Goro KK, et al.[[37](#_ENREF_37)] \| 2019 \| Cross Sectional \| Oromia \| patient interview, record review \| JU \| age \| 2.01 \| 1.1-5 \| 8.5 \| \| Tesfe D, et al.[[38](#_ENREF_38)] \| 2022 \| Cross Sectional \|  \| record review \| Not funded \| age \| 5.74 \| 3.05-10 \| 9 \| \| Fiseha T, etal.[[39](#_ENREF_39)] \| 2020 \| Cross Sectional \| Amhara \| patient interview \| WU \| age \| 2.48 \| 1.13-5.43 \| 8 \| | 9 |
| Results of syntheses | 20a | The risk of bias was assed using Begg’s and/or Egger’s test. | 8 |
|  | 20b | The search strategy retrieved 1145 both published and unpublished original articles. After the removal of duplicate articles, 127 articles remained. Following further screening, about 19 articles were assessed for eligibility, of which three articles were excluded because of reporting without the outcome of interest, poor quality, and not full text.  The finding of this review study revealed that, age of diabetes (AOR= 1.11, 95% CI: 1.03,120, I^2^= 0.0%, P= 0.488), duration of diabetic illness (AOR=1.23, 95% CI= 1.05, 1.45, I^2^= 0.0%, p=0.567), poor glycemic control (AOR=2.57, 95%CI: 1.07, 6.14; I^2^ = 0.0%, p=0.996), and co-morbid hypertension (AOR=4.03, 95%CI: 2.00-8.12, I^2^ = 0.0%, p- 0.964). | 11 |
|  | 20c | The overall heterogeneity test (**I^2^**) on the effect of co-morbid HTN was 0.0% with a p-value < 0.996, using a random effect model to adjust observed variability. This indicates there is no variability across the studies. | 12 |
|  | 20d | The publication bias test result of Egger’s test p-value is 0.07and the funnel plot test looks symmetrical (**Fig. 9**) indicating there is no publication bias. | 10 |
| Reporting biases | 21 | This study hasn’t publication bias as shown by the inverted funnel plot which symmetrically distributed (**Fig** 3). Begg's test and Egger’s test were done with p >0.494 which showed that the absence of publication bias. | 12 |
| Certainty of evidence | 22 | In this systematic and meta-analysis study, co-morbid HTN, poor glycemic control, and longer duration of diabetes illness were found to be the determinant factors of diabetic nephropathy. | 15 |
| **DISCUSSION** | | |  |
| Discussion | 23a | This systematic review and meta-analysis study identifies the important risk factors of diabetic nephropathy. The finding of this study revealed that advanced age (≥55 years) is the risk factors of diabetic nephropathy. The finding of the study is supported by the study conducted in Italy depicted that diabetic patients with advanced age are strongly associated with low estimated glomerular filtration rate[[46](#_ENREF_46)]. This is due to the fact that advanced age results in stiffness the blood vessels eventually lead to cardiovascular and renal complications[[47](#_ENREF_47)]. Furthermore, aging can predispose to various co-morbidities[[48](#_ENREF_48)].  Duration of diabetic illness is the determinant factor for the development of diabetic nephropathy. Patients with longer duration with diabetic illness (≥10 years) are at risk of developing diabetic nephropathy. This is consistent with the The finding also depicts glycemic control status is the determinant factors of diabetic nephropathy. Patients with poor glycemic control are at risk for developing diabetic nephropathy. This is supported by the review study [[49](#_ENREF_49)]. Over time, poorly controlled diabetes can cause damage to blood vessel clusters in the kidneys that filters the wastes[[50](#_ENREF_50)]. Furthermore, co-morbid hypertension is a determinant factor of diabetic kidney disease. The result of this study is supported by the study conducted in Sub Saharan Africa, which stated that the risk of diabetic nephropathy is higher in diabetic patients with hypertension compared with non hypertensive patients [[51](#_ENREF_51)]. This is because of renal arterial sclerosis, and metabolic abnormalities derived from diabetes affects the renal function [[52](#_ENREF_52)]. The elevated hypertension directly transmitted to macro vascular and gromeruli. This leads to glomerular hypertension and activation of mediators that induce inflammation, fibrosis, and further injury of the glomeruli [[53](#_ENREF_53)]. High blood pressure can cause further kidney damage by increasing the pressure in the delicate system of the kidney[[54](#_ENREF_54)]. | 13 |
|  | 23b | The finding of this study has important limitations; 1) the study only contains observational study designs; 2) restricted only for English language; 3)focused on determinants factors of diabetic kidney disease but not its prevalence; 4) the authors include both type 1 and/or type 2 diabetic patients; and 5) the authors used specific factors. Therefore, the authors recommended further research that includes other than observational study design; conduct on type 1 and type 2 diabetes patients separately; and other predictors of diabetic nephropathy need to be studied. | 14 |
|  | 23c | The study has an implication for policymakers and clinicians to plan and implement possible interventions to prevent the occurrence and severe outcome of diabetic nephropathy. | 14 |
|  | 23d | The finding of this systematic review and meta analysis study revealed that, diabetic patients with advanced age, longer duration of diabetic illness, poorglycemic control status, and having co-morbid hypertension were found the determinant factors of diabetic nephropathy. Therefore, Treatment of comorbid hypertension and blood glucose, and regular screening of renal function should be implemented to reduce the occurrence of diabetic kidney diseases. Furthermore, health care workers should give due attention for diabetic patients with advanced age, longer duration of diabetic illness, poor glycemic control, and co-morbid diabetes. | 15 |
| **OTHER INFORMATION** | | |  |
| Registration and protocol | 24a | The review protocol has been registered in the international prospective register of systematic reviews (PROSPERO) with registration number PROSPERO**:** CRD42023434547. |  |
|  | 24b | The review protocol can be accessed via online databases. |  |
|  | 24c | Further amendments may /not needed. |  |
| Support | 25 | The Authors did not receive any fund for this particular study. |  |
| Competing interests | 26 | There is no competing of interest. |  |
| Availability of data, code and other materials | 27 | The data extracted were analysed and included in the result. |  |

*From:* Page MJ, McKenzie JE, Bossuyt PM, Boutron I, Hoffmann TC, Mulrow CD, et al. The PRISMA 2020 statement: an updated guideline for reporting systematic reviews. BMJ 2021;372:n71.doi: 10.1136/bmj.n71

For more information, visit:<http://www.prisma-statement.org/>
