# Supplementary material for: Determinants of diabetic nephropathy among diabetic patients in Ethiopia: Systematic review and meta-analysis
Source: PLoS One. 2024 Feb 2;19(2):e0297082. doi: 10.1371/journal.pone.0297082 (PMC10836702; doi:10.1371/journal.pone.0297082)
Supplement: S2 File — (DOCX) [file pone.0297082.s002.docx]

**Table 1: Study characteristics for the age of diabetic patients in Ethiopia**

| **Authors** | **year** | **study**  **design** | **Region** | **data**  **collection** | **Funding source** | **Factors** | **Odds ratio** | **Log OR** | **LBCI** | **Log**  **LBCI** | **UBCI** | **Log**  **UBCI** | **SeLogOR** | **quality score** |
| --- | --- | --- | --- | --- | --- | --- | --- | --- | --- | --- | --- | --- | --- | --- |
| Damtie S, et al.[[33](#_ENREF_33)] | 2018 | Cross sectional | Amhara | patient interview,  record review | Not funded | age | 5.239 | 1.656131 | 2.255 | 0.81315 | 12.175 | 2.499385 | 1.65251 | 9 |
| Fiseha T, et al.[[34](#_ENREF_34)] | 2014 | Cross sectional | SNNP | patient interview | Not reported | age | 5.3 | 1.667707 | 1.81 | 0.593327 | 15.56 | 2.744704 | 2.108349 | 8 |
| Dinku B, et al.[[35](#_ENREF_35)] | 2022 | Cross Sectional | Oromia | patient interview,  record review | St PMMC | age | 2.17 | 0.774727 | 1.09 | 0.086178 | 4.31 | 1.460938 | 1.347265 | 8.5 |
| Hintsa S, et al.[[18](#_ENREF_18)] | 2017 | Case control | Tigray | record review | Not funded | age | 1.037 | 0.036332 | 1.01 | 0.00995 | 1.064 | 0.062035 | 0.051043 | 7 |
| Mulu GB, et al.[[36](#_ENREF_36)] | 2023 | Cross Sectional | Amhara | patient interview | Not reported | age | 4.1 | 1.410987 | 2.2 | 0.788457 | 7.7 | 2.04122 | 1.227708 | 7.5 |
| Zemichael TM, et al.[[9](#_ENREF_9)] | 2020 | Case control | Tigray | patient interview | Not reported | age | 1.19 | 0.173953 | 1.16 | 0.14842 | 1.23 | 0.207014 | 0.057422 | 8 |
| Abdulkadir M, et al.[[37](#_ENREF_37)] | 2022 | Cross Sectional | Addis Ababa | patient interview | Not funded | age | 5.8 | 1.757858 | 1.5 | 0.405465 | 21 | 3.044522 | 2.586276 | 8 |
| Adem M, et al.[[38](#_ENREF_38)] | 2017 | Cross Sectional | Oromia | patient interview | Not funded | age | 3.02 | 1.105257 | 1.55 | 0.438255 | 5.9 | 1.774952 | 1.309963 | 7.5 |
| Goro KK, et al.[[39](#_ENREF_39)] | 2019 | Cross Sectional | Oromia | patient interview,  record review | JU | age | 2.01 | 0.698135 | 1.1 | 0.09531 | 5 | 1.609438 | 1.483845 | 8.5 |
| Tesfe D, et al.[[40](#_ENREF_40)] | 2022 | Cross Sectional |  | record review | Not funded | age | 5.74 | 1.747459 | 3.05 | 1.115142 | 10 | 2.302585 | 1.163695 | 9 |
| Fiseha T, etal.[[41](#_ENREF_41)] | 2020 | Cross Sectional | Amhara | patient interview | WU | age | 2.48 | 0.908259 | 1.13 | 0.122218 | 5.43 | 1.691939 | 1.538327 | 8 |

Notes:- **CI-Confidence interval; JU-** Jimma University**; St PMMC-** Sight Paulos Millennium Medical College**; SNNP**-Southern Nations, Nationalities and Peoples; **WU-** Wollo University

**Table 2: Study characteristics for the duration of diabetic illness among diabetic patients in Ethiopia**

| **Authors** | **year** | **study**  **design** | **Region** | **data**  **collection** | **Funding**  **source** | **Factors** | **Odds**  **ratio** | **LogOR** | **LBCI** | **LogLBCI** | **UBCI** | **LogUBCI** | **SeLogOR** | **quality score** |
| --- | --- | --- | --- | --- | --- | --- | --- | --- | --- | --- | --- | --- | --- | --- |
| Alemu H, et al.[[12](#_ENREF_12)] | 2020 | Cross Sectional | Amhara | patient interview, record review | not reported | duration  of diabetic  illness | 3.2 | 1.163151 | 2 | 0.693147 | 7 | 1.94591 | 1.227708 | 9 |
| Bekele MM, et al.[[42](#_ENREF_42)] | 2016 | Cross Sectional | Addis Ababa | patient interview, record review | not reported | duration of diabetic illness | 1.45 | 0.371564 | 0.73 | -0.31471 | 2.88 | 1.05779 | 1.345051 | 8 |
| Damtie S, et al.[[33](#_ENREF_33)] | 2018 | Cross Sectional | Amhara | patient interview, record review | Not funded | duration of diabetic illness | 3.38 | 1.217876 | 1.393 | 0.33146 | 8.198 | 2.10389 | 1.736982 | 9 |
| Fiseha T, et al.[[34](#_ENREF_34)] | 2014 | Cross Sectional | SNNP | Patient interview | not reported | duration of diabetic illness | 4.08 | 1.406097 | 1.7 | 0.530628 | 9.77 | 2.279316 | 1.713714 | 8 |
| Hintsa S, et al.[[18](#_ENREF_18)] | 2017 | Case control | Tigray | Record review | not funded | duration of diabetic illness | 1.13 | 0.122218 | 1.077 | 0.074179 | 1.18 | 0.165514 | 0.089508 | 7 |
| Taderegew MM, et al.[[43](#_ENREF_43)] | 2020 | Cross Sectional | Amhara | patient interview, record review | not funded | duration of diabetic illness | 3.38 | 1.217876 | 1.45 | 0.371564 | 7.82 | 2.056685 | 1.651419 | 8 |
| Zemichael TM, et al.[[9](#_ENREF_9)] | 2020 | Case control | Tigray | patient interview | not reported | duration of diabetic illness | 1.83 | 0.604316 | 1.62 | 0.482426 | 2.06 | 0.722706 | 0.235474 | 8 |
| Adem M, et al.[[38](#_ENREF_38)] | 2017 | Cross Sectional | Oromia | patient interview | not reported | duration of diabetic illness | 4.44 | 1.490654 | 2.3 | 0.832909 | 8.57 | 2.148268 | 1.289051 | 7.5 |
| Tesfe D, et al.[[40](#_ENREF_40)] | 2022 | Cross Sectional | Amhara | record review | Not funded | duration of diabetic illness | 3.37 | 1.214913 | 1.86 | 0.620576 | 6.1 | 1.808289 | 1.163958 | 9 |
| Fiseha T, et al.[[41](#_ENREF_41)] | 2020 | Cross Sectional | Amhara | patient interview | WU | duration of diabetic illness | 5.16 | 1.640937 | 1.13 | 0.122218 | 12.51 | 2.526528 | 2.356224 | 8 |

**Notes:- CI-** Confidence interval; **SNNP**-Southern Nations, Nationalities and Peoples

**Table 3: Study characteristics for patients with poor glycemic status among diabetic patients in Ethiopia**

| **Authors** | **year** | **study**  **design** | **Region** | **data**  **collection** | **Funding source** | **Factors** | **Odds ratio** | **LogOR** | **LBCI** | **LogLBCI** | **UBCI** | **LogUBCI** | **SeLogOR** | **quality score** |
| --- | --- | --- | --- | --- | --- | --- | --- | --- | --- | --- | --- | --- | --- | --- |
| Aberra T, et al.[[16](#_ENREF_16)] | 2022 | Cross-sectional | Addis Ababa | Interviewer administer | not reported | PGC | 1.23 | 0.207014 | 0.667 | -0.40497 | 2.276 | 0.82242 | 1.202837 | 9 |
| Fiseha T, et al.[[34](#_ENREF_34)] | 2014 | Cross sectional | SNNP | patient interview | not reported | PGC | 4.65 | 1.536867 | 1.69 | 0.524729 | 12.76 | 2.546315 | 1.981155 | 8 |
| Dinku H, et al.[[35](#_ENREF_35)] | 2022 | Cross sectional | Oromia | patient intrview, record review | St.PHMMC | PGC | 1.37 | 0.314811 | 0.75 | -0.28768 | 2.49 | 0.912283 | 1.175965 | 7.5 |
| Hintsa S, et al.[[18](#_ENREF_18)] | 2017 | Case control | Tigray | record review | not funded | PGC | 2.71 | 0.996949 | 1.49 | 0.398776 | 4.95 | 1.599388 | 1.176599 | 7 |
| Mulu GB, et al.[[36](#_ENREF_36)] | 2023 | Cross Sectional | Amhara | patient interview | not reported | PGC | 2.5 | 0.916291 | 1.5 | 0.405465 | 4.1 | 1.410987 | 0.985411 | 7.5 |
| Taderegew MM, et al.[[43](#_ENREF_43)] | 2020 | Cross Sectional | Amhara | patient interview, Record review | not funded | PGC | 2.82 | 1.036737 | 1.13 | 0.122218 | 7.05 | 1.953028 | 1.794194 | 8 |
| Zemichael TM, et al.[[9](#_ENREF_9)] | 2020 | Case control | Tigray | patient interview | not reported | PGC | 3.27 | 1.18479 | 1.31 | 0.270027 | 8.31 | 2.11746 | 1.810484 | 8 |
| Adem M, et al.[[38](#_ENREF_38)] | 2017 | Cross Sectional | Oromia | patient interview | not funded | PGC | 7.24 | 1.979621 | 2.16 | 0.770108 | 24.22 | 3.187179 | 2.368729 | 7.5 |
| Nibret E, et al.[[44](#_ENREF_44)] | 2020 | cohort | SNNPR | record review | not reported | PGC | 9.09 | 2.207175 | 4 | 1.386294 | 20 | 2.995732 | 1.577249 | 8.5 |
| Fiseha T, et al.[[41](#_ENREF_41)] | 2020 | Cross Sectional | Amhara | patient interview | WU | PGC | 2.58 | 0.947789 | 1.19 | 0.173953 | 5.61 | 1.724551 | 1.519585 | 8 |

**Notes: CI-** Confidence interval; **PGC**- poor glycemic control; ; **St.PHMMC**: Sight Paulos hospital Millennium Medical College; **SNNP**-Southern Nations, Nationalities and Peoples; **WU**: Wollo University

**Table 4: Study characteristics for patients with elevated systolic blood pressure among diabetic patients in Ethiopia**

| **Authors** | **year** | **study**  **design** | **Region** | **data**  **collection** | **Funding**  **source** | **Factors** | **Odds**  **ratio** | **LogOR** | **LBCI** | **LogLBCI** | **UBCI** | **LogUBCI** | **SeLogOR** | **quality**  **score** |
| --- | --- | --- | --- | --- | --- | --- | --- | --- | --- | --- | --- | --- | --- | --- |
| Dinku B, et al.[[35](#_ENREF_35)] | 2022 | Cross sectional | Oromia | patient interview, record review | St.PHMMC | elevated  SBP | 3.2 | 1.163151 | 1.36 | 0.307485 | 7.51 | 2.016235 | 1.674576 | 8.5 |
| Hintsa S, et al.[[18](#_ENREF_18)] | 2017 | Case control | Tigray | Record review | not funded | elevated  SBP | 2.78 | 1.022451 | 1.69 | 0.524729 | 4.58 | 1.521699 | 0.977031 | 7 |
| Mulu GB, et al.[[36](#_ENREF_36)] | 2023 | Cross Sectional | Amhara | patient interview | not reported | elevated  SBP | 1.9 | 0.641854 | 1 | 0 | 3.4 | 1.223775 | 1.1993 | 7.5 |
| Taderegew MM, et al.[[43](#_ENREF_43)] | 2020 | Cross Sectional | Amhara | patient interview, record review | not funded | elevated SBP | 0.78 | -0.24846 | 0.49 | -0.71335 | 1.07 | 0.067659 | 0.765388 | 8 |
| Alemu H, et al.[[12](#_ENREF_12)] | 2020 | Cross Sectional | Amhara | patient interview, record review | not reported | elevated SBP | 6.0 | 1.791759 | 4 | 1.386294 | 22 | 3.091042 | 1.670653 | 8.5 |
| Adem M, et al.[[38](#_ENREF_38)] | 2017 | Cross Sectional | Oromia | patient interview | not funded | elevated SBP | 2.19 | 0.783902 | 1.12 | 0.113329 | 4.28 | 1.453953 | 1.313812 | 7.5 |
| Tesfe D, et al.[[40](#_ENREF_40)] | 2022 | Cross Sectional | Amhara | record  review | not funded | elevated SBP | 6.33 | 1.8453 | 3.34 | 1.205971 | 11.99 | 2.484073 | 1.25254 | 9 |
| Adem M, et al.[[45](#_ENREF_45)] | 2021 | Cross sectional | Amhara | record review | not reported | elevated SBP | 0.997 | -0.003 | 0.594 | -0.52088 | 1.72 | 0.542324 | 1.041936 | 8 |

**Notes: - CI-**Confidence interval; **SBP-**Systolic blood pressure; **St.PHMMC**: Sight Paulos hospital Millennium Medical College

**Table 5: Study characteristics for patients’ co-morbid hypertension among diabetic patients in Ethiopia**

| **Authors** | **year** | **study**  **design** | **Region** | **data**  **collection** | **Funding**  **source** | **Factors** | **Odds ratio** | **LogOR** | **LBCI** | **LogLBCI** | **UBCI** | **LogUBCI** | **SeLogOR** | **quality score** |
| --- | --- | --- | --- | --- | --- | --- | --- | --- | --- | --- | --- | --- | --- | --- |
| Aberra T, et al.[[16](#_ENREF_16)] | 2022 | Cross Sectional | Addis Ababa | patient interview | not reported | Co-HTN | 1.37 | 0.314811 | 0.865 | -0.14503 | 2.169 | 0.774266 | 0.900906 | 8.5 |
| Adem M, et al.[[45](#_ENREF_45)] | 2021 | Cross Sectional | Amhara | patient interview,  record review | not reported | Co-HTN | 2.279 | 0.823737 | 1.025 | 0.024693 | 5.067 | 1.622749 | 1.566095 | 8 |
| Alemu H, et al.[[12](#_ENREF_12)] | 2020 | Cross Sectional | Amhara | patient interview  record review | not reported | Co-HTN | 8.2 | 2.104134 | 2 | 0.693147 | 23 | 3.135494 | 2.101 | 8.5 |
| Bekele MM, et al.[[42](#_ENREF_42)] | 2016 | Cross Sectional | Addis Ababa | patient interview,  record review | not reported | Co-HTN | 1.16 | 0.14842 | 0.81 | -0.21072 | 3.26 | 1.181727 | 1.364599 | 7 |
| Damtie S, et al.[[33](#_ENREF_33)] | 2018 | Cross Sectional | Amhara | patient interview,  record review | not funded | Co-HTN | 4.51 | 1.506297 | 2.266 | 0.818016 | 8.977 | 2.194666 | 1.349117 | 9 |
| Dinku B, et al.[[35](#_ENREF_35)] | 2022 | Cross Sectional | Oromia | patient interview,  record review | St.PHMMC | Co-HTN | 4.89 | 1.587192 | 1.93 | 0.65752 | 12.4 | 2.517696 | 1.822973 | 8.5 |
| Taderegew MM ,et al.[[43](#_ENREF_43)] | 2020 | Cross Sectional | Amhara | patient interview,  record review | not funded | Co-HTN | 3.12 | 1.137833 | 1.51 | 0.41211 | 6.45 | 1.86408 | 1.422931 | 8 |
| Zemichael TM, et al.[[9](#_ENREF_9)] | 2020 | Case control | Tigray | patient interview | not reported | Co-HTN | 6.44 | 1.862529 | 4.46 | 1.495149 | 9.28 | 2.227862 | 0.718059 | 8 |
| Adem M, et al.[[38](#_ENREF_38)] | 2017 | Cross Sectional | Oromia | patient interview | not funded | Co-HTN | 5.62 | 1.726332 | 2.81 | 1.033184 | 11.23 | 2.418589 | 1.357696 | 7.5 |
| Tesfe D ,et al.[[40](#_ENREF_40)] | 2022 | Cross Sectional | Amhara | record review | not funded | Co-HTN | 4.85 | 1.578979 | 2.07 | 0.727549 | 11.3 | 2.424803 | 1.663309 | 9 |
| Nibret E, et al.[[44](#_ENREF_44)] | 2020 | retro cohort | SNNPR | Record review | not reported | Co-HTN | 12.5 | 2.525729 | 8.33 | 2.119863 | 25 | 3.218876 | 1.077032 | 8.5 |
| Fiseha T, et al.[[41](#_ENREF_41)] | 2020 | Cross Sectional | Amhara | patient interview | WU | Co-HTN | 3.37 | 1.214913 | 1.45 | 0.371564 | 7.86 | 2.061787 | 1.656419 | 8 |

**Notes:-CI-**Confidence interval; **Co-HTN**-Comorbid hypertension; **St.PHMMC**: Sight Paulos hospital Millennium Medical College; **SNNP**-Southern Nations, Nationalities and Peoples; **WU**: Wollo University
