# Supplementary material for: Determinants of diabetic nephropathy among diabetic patients in Ethiopia: Systematic review and meta-analysis
Source: PLoS One. 2024 Feb 2;19(2):e0297082. doi: 10.1371/journal.pone.0297082 (PMC10836702; doi:10.1371/journal.pone.0297082)
Supplement: Attachment — Submitted filename: comments to authhor.docx [file pone.0297082.s003.docx]

The author comes up with nice title but similar with previous work published in similar study area and similar associated factors (https://www.hindawi.com/journals/ijn/2020/8890331/
