# Supplementary material for: Determinants of diabetic nephropathy among diabetic patients in Ethiopia: Systematic review and meta-analysis
Source: PLoS One. 2024 Feb 2;19(2):e0297082. doi: 10.1371/journal.pone.0297082 (PMC10836702; doi:10.1371/journal.pone.0297082)
Supplement: Attachment — Submitted filename: Response to Reviewers.docx [file pone.0297082.s004.docx]

**Response to Reviewers**

| **S.no** | **Reviewer #1 questions & comments** | **Responses** | | **Remark** | |
| --- | --- | --- | --- | --- | --- |
| 1. | Your tile is timely and uncover | Thank you very much | |  | |
| 2 | Abstract is advisable to be one page | It is summarized to one page | | see abstract section  Line 25-46 | |
| 3 | The study population should be included in the method and in the abstract as well. | it is included | | Abstract;  Line 31-32  Method;  Line: 99-101 | |
| 4 | Line 68-70: is the prevalence global? Regional? Or country level? | It was the global prevalence, but modified based on the objective of the study. | |  | |
| 5 | Line 75: what do you mean; There was no diabetic nephropathy (DN) without being diagnosed with diabetes? | This is to say; diabetic nephropathy is the only disorder for diabetic patients but not for non diabetic ones. | | Line 55-56 | |
| 6 | Your introduction should focus in your title. In some of your paragraphs seems that you are studying on DM rather than DN. | It is modified and corrected based on the objective of the study. | | Introduction section | |
| 7 | Line 181: what is the outcome of interest? What is poor quality? Define them | The definition of Outcome of interest is stated at outcome measurement section. Poor quality of article was considered when the Newcastle Ottawa scale is less than five. | | Outcome interest;  Line: 118-120  poor quality Line: 143-144 | |
| 8 | Age and duration of diabetes are as continuous in some of the studies and categorized in the other studies. How do you pooled it their respective odds ratios? | The authors pooled the odds ratio of age and duration of diabetic illness using the crosstabs. It was calculated using the frequencies distributed in the crosstabs of the included factors. | |  | |
| 9 | Discussion: It is good to compare with other studies, but, your implication also needed as you did it for duration of illness through it not detail. | It is edited and corrected. | | See discussion and implication section | |
| **Reviewer #2 comments** | | | | | |
| 1 | The author did not acknowledge previous work similar with this current submission or the reason for repetition of this study. | **The former study** | **The current study** | |  |
|  |  | - The former study focused on the burden/ prevalence of chronic kidney disease and the specific factors among diabetic patients. - Chronic kidney disease is a broad term which indicates the structural damage of the kidney. - It may be macro or microvascular complication of the kidney’s structure. - The factors used in the former study are similar within diabetic and non diabetic patients of chronic kidney disease patients. | - The current study (diabetic nephropathy) is focused on determinant factors of diabetic nephropathy. - Diabetic Nephropathy is the microvascular complication which mainly affects the renal vasculatures. - Diabetic nephropathy is unique to diabetic population - The factors may be similar to the factors of the former study but its contribution to cause diabetic nephropathy is different. | |  |
| **Editor comments** | |  | | |  |
| 1 | Please justify the reason why you interested in the title | Nowadays, diabetic associated complication is the leading causes of mortality, morbidity, and economic impact, but its determinant factors are not adequately investigated.  In addition, different evidences depict that diabetic patients are at risk of end stage renal disease. But the evidences are inconsistent across the studies. | | |  |
| 2 | Focus on the objective when you frame your introduction section | It is modified based on the objective of the study. | | | see introduction section |
| 3 | You need to revise the discussion section | It is edited and corrected. | | | see the discussion section |
