# Supplementary material for: Determinants of diabetic nephropathy among diabetic patients in Ethiopia: Systematic review and meta-analysis
Source: PLoS One. 2024 Feb 2;19(2):e0297082. doi: 10.1371/journal.pone.0297082 (PMC10836702; doi:10.1371/journal.pone.0297082)
Supplement: Attachment — Submitted filename: comments to author DN.docx [file pone.0297082.s005.docx]

Comments to author

- The author comes up with nice title but similar with previous work published in similar study area (<https://www.hindawi.com/journals/ijn/2020/8890331/>, which needs either brief explanation for the repetition of this study , mostly meta-analysis experts recommends it shouldn’t be conducted if there is published study between five years.
- In abstract part of result section sample size for each included study under predictors should be mentioned
- In abstract part of conclusion statement recommendation should be added…..the finding of the study revealed that diabetic patients with advanced age, longer duration of diabetic illness, poor glycemic control status, and co-morbid hypertension were found the determinant factors of DN……so what?????????
- The authors mentioned they were used PRISMA 2020 for reporting but they are not followed
- They authors didn’t do comprehensive search, they missed studies for example <https://www.dovepress.com/time-to-diabetic-nephropathy-and-its-predictors-among-diabetic-patient-peer-reviewed-fulltext-article-IJNRD#:~:text=The%20mean%20time%20to%20develop,being%20hypertensive%20were%20the%20predictors>.
- Under result part unnecessary information was extracted like funding , data collection method and the main variable( it is already mentioned in the heading)
- In result part putting result is enough no need of description like Diabetic patients with older age are 1.11 times more likely to develop DN compared to those diabetic patients with younger age (AOR = 1.11, 95% CI: 1.03,120, I2 202 = 0.0%, P = 0.488) (Fig. 203 )….applicable for all
- In discussion part- the author discussed the finding with unrelated setting, so should be discussed with nearly related setting in different factors like culture, economic status n others)
