# Supplementary material for: Determinants of diabetic nephropathy among diabetic patients in Ethiopia: Systematic review and meta-analysis
Source: PLoS One. 2024 Feb 2;19(2):e0297082. doi: 10.1371/journal.pone.0297082 (PMC10836702; doi:10.1371/journal.pone.0297082)
Supplement: Attachment — Submitted filename: Response to Reviewers.docx [file pone.0297082.s007.docx]

**Response to Reviewers**

| **S.No** | **comments to Authors** | **Responses** | **Remark** |
| --- | --- | --- | --- |
| **I** | **ACADEMIC EDITOR comments** | | |
|  | Please respond to the comments given by **reviewer #2**. In addition, abstract section is too shallow please make it comprehensive and informative | Thank you, the concerns raised by reviewer #2 was corrected, and the abstract section was edited and corrected. | See the abstracted section |
| **II** | **Reviewer #2 questions & comments** |  |  |
| **1** | Is the manuscript presented in an intelligible fashion and written in standard English?  PLOS ONE does not copyedit accepted manuscripts, so the language in submitted articles must be clear, correct, and unambiguous. Any typographical or grammatical errors should be corrected at revision, so please note any specific errors here.  Reviewer #2: No | The whole manuscript was edited and corrected by professional language experts. | See the whole manuscript |
| **2** | It is already previously published work, if it has a justification for the repetition of the study, it is worthy if published. | Diabetic nephropathy and chronic kidney disease are common disorders in diabetic population, but diabetic nephropathy is the most common renal disorder among diabetic population only, whereas CKD occurs among diabetes and/or non diabetes. Diabetic nephropathy is purely associated with the presence of diabetes, but CKD may not associate with diabetes. We don’t know which comes first diabetes or CKD. As per our review, there was no similar published study on diabetic nephropathy. There may be published articles on chronic kidney disease but there is a difference in between. Diabetic nephropathy refers generally to the damage to the kidneys caused by diabetes, where as chronic kidney disease has a specific clinical definition and may be caused by diabetes or by other diseases. But DN is prone for diabetic patients.  According to studies one in five people need treatment for diabetic nephropathy.(**DiABETES UK**) |  |
| **3** | PLOS authors have the option to publish the peer review history of their article ([what does this mean?](https://journals.plos.org/plosone/s/editorial-and-peer-review-process#loc-peer-review-history)). If published, this will include your full peer review and any attached files.  If you choose “no”, your identity will remain anonymous but your review may still be made public. | Yes, it is possible to be included in the publication |  |
| 4. | The author comes up with nice title but similar with previous work published in similar study area (<https://www.hindawi.com/journals/ijn/2020/8890331/>, which needs either brief explanation for the repetition of this study , mostly meta-analysis experts recommends it shouldn’t be conducted if there is published study between five years. | The former study focused on Chronic Kidney disease but the current one focused on diabetic nephropathy. They are clinically different. The factors used in CKD may similar with the current study but the mechanism of diabetic nephropathy occurrence is different from CKD. All in all, diabetic nephropathy and CKD are not the same disorder. That is way the authors tried to investigate the prevalence and its predictors of diabetic nephropathy.  **For more detail please see the above descriptions QNo #2** |  |
| 5. | In abstract part of result section sample size for each included study under predictors should be mentioned | It is incorporated |  |
| 6. | In abstract part of conclusion statement recommendation should be added…..the finding of the study revealed that diabetic patients with advanced age, longer duration of diabetic illness, poor glycemic control status, and co-morbid hypertension were found the determinant factors of DN……so what????????? | It is edited and corrected. The recommendation is included | see conclusion section of the abstract |
| 7. | The authors mentioned they were used PRISMA 2020 for reporting but they are not followed | It is revised and corrected. There are a number of flow diagrams but the authors used “[PRISMA 2020 flow diagram for new systematic reviews which included searches of databases and registers only](http://www.prisma-statement.org/documents/PRISMA_2020_flow_diagram_new_SRs_v1.docx)” | Fig 1. |
| 8. | They authors didn’t do comprehensive search, they missed studies for example <https://www.dovepress.com/time-to-diabetic-nephropathy-and-its-predictors-among-diabetic-patient-peer-reviewed-fulltext-article-IJNRD#:~:text=The%20mean%20time%20to%20develop,being%20hypertensive%20were%20the%20predictors> | The authors used standard search strategies to include published/unpublished articles **up to June 2023.** The article you mentioned was included in the study but it was excluded due to poor quality i.e. the effect measure was used in this study was Adjusted hazard ratio/AHR/ but the author used the effect measure of odds ratio/OR/, so the authors calculated the effect measure from crosstabs which gives odds ratio of 19.46 times for co-morbid hypertension which is exaggerated/unreliable estimate so the authors excluded it from the study. |  |
| 9. | Under result part unnecessary information was extracted like funding , data collection method and the main variable( it is already mentioned in the heading) | it is edited and removed | see table 1-5 |
| 10. | In result part putting result is enough no need of description like Diabetic patients with older age are 1.11 times more likely to develop DN compared to those diabetic patients with younger age (AOR = 1.11, 95% CI: 1.03,120, I2 202 = 0.0%, P = 0.488) (Fig. 203 )….applicable for all | **It is edited and revised.** | see result section |
| 11 | In discussion part- the author discussed the finding with unrelated setting, so should be discussed with nearly related setting in different factors like culture, economic status n others) | Ok, thank you. We tried to incorporate your concerns but the study focused on determinant factors as a result it must show the interaction of the factors with the dependent variable/diabetic nephropathy. If it the study is prevalence/magnitude it must incorporate your concerns. | Line: 275-276 280-282 |
